# Overview in Electrochemical and Electrical Biosensors for Determining Blood Protein Biomarkers of Alzheimer’s Disease

**DOI:** 10.3390/bios16080426

**Published:** 2026-08-06

**Authors:** Fengli Gao, Lin Liu, Junyue Li, Shaoyang Chen, Xinyao Yi

**Affiliations:** 1 Henan Province of Key Laboratory of New Optoelectronic Functional Materials, College of Chemistry and Chemical Engineering, Anyang Normal University, Anyang 455000, China; flgao@aynu.edu.cn (F.G.); 18338016065@163.com (J.L.); 18137297039@163.com (S.C.); 2 College of Chemistry and Chemical Engineering, Central South University, Changsha 410083, China

**Keywords:** Alzheimer’s disease, blood biomarkers, proteins, electrochemical biosensors, electrical biosensors

## Abstract

Early diagnosis of Alzheimer’s disease (AD) can facilitate the establishment and implementation of therapeutic interventions. The currently used diagnosis methods for AD mainly include cerebrospinal fluid analysis and positron emission tomography imaging. Due to their high invasiveness, high cost, and limited accessibility, these technologies are difficult to meet the needs of large-scale population screening, grading diagnosis, and treatment, thereby limiting the popularization of early diagnosis of AD. The detection of blood biomarkers has become an important breakthrough in early screening and diagnosis of different diseases due to its non-invasive, low-cost, and easy-to-operation advantages. Recently, blood proteins such as amyloid-beta (Aβ), total and phosphorylated Tau, light chain neurofilaments (NFL), and glial fibrillary acidic protein (GFAP) have been considered promising biomarkers for the diagnosis of AD. However, there is currently no effective, minimally invasive, and easily accessible detection method for clinical diagnosis and risk prediction of AD. Electrochemical and electrical biosensors are highly sensitive, simple, fast, and cost-effective analytical tools for disease monitoring, drug development, and target detection. In this work, we comprehensively and systematically overview the progress of various electrochemical and electrical techniques for determining AD-related blood protein biomarkers, mainly including electrochemistry, electrochemiluminescence, photoelectrochemistry, quartz crystal microbalance, field-effect transistor, and organic electrochemical transistor. This work can provide guidance for researchers to develop novel electrochemical and electrical biosensors for early and accurate diagnosis of AD.

## 1. Introduction

Alzheimer’s disease (AD) is the most common form of dementia in the elderly. It is characterized by the deposition of amyloid-beta (Aβ) peptide senile plaques in brain tissue and phosphorylated Tau (p-Tau) proteins in the neurofibrils of brain neurons [1]. Before 2000, the clinical diagnosis of AD mainly relied on clinical symptoms, evaluation of cognitive function assessment, and exclusion of other possible causes of dementia. A definitive diagnosis could only be made by postmortem examination to confirm the presence of Aβ deposits and neurofibrillary tangles in brain. Later, structural magnetic resonance imaging (MRI) of the hippocampus became an important method for evaluating the progression of AD patients. Recently, novel imaging techniques based on positron emission tomography (PET) have been approved to monitor Aβ deposition in the brain for clinical diagnosis [2]. Although the exact molecular mechanisms leading to AD remain highly controversial, substantial evidences indicate that the age-related plaques of Aβ in brain tissue can be found much earlier than the deposition of p-Tau in neurofibrillary tangles and the onset of clinical symptoms of AD [3,4]. Another evidence supporting Aβ as a pathogenic factor and demonstrating its core role in the pathogenesis of disease comes from the study on the familial form of AD. Therefore, Aβ has become the most important biomarker and target for AD diagnosis and treatment. Recent clinical trials aimed at inhibiting Aβ production or clearing its aggregates and moderating AD patients through antibody drugs have failed to achieve the expected therapeutic effects [5]. One possible reason for the failure is that anti-Aβ therapies cannot rescue neurons and synapses that have been damaged by toxic Aβ species. In addition, most of the clinical trials mainly recruit individuals who have not yet shown symptoms of AD, rather than patients with symptomatic AD or mild cognitive impairment [6]. Therefore, reliable biomarkers are needed for rapid and early diagnosis of AD before the onset of dementia, while minimizing invasiveness to the greatest extent possible.

Biomarkers in cerebrospinal fluid (CSF) can directly reflect the biochemical changes of AD brain due to its contact with the brain’s extracellular space. Since the 1990s, research has mainly focused on the key pathogenic biomolecules of AD in CSF, including Aβ, total Tau, and p-Tau, which are now identified as the reliable biomarkers for AD diagnosis [7]. According to the concentration changes of these biomarkers, AD patients can be identified with an accuracy exceeding 80%. However, the collection of CSF samples requires invasive and high-risk procedures, making it unsuitable for screening asymptomatic individuals. Researches on CSF biomarkers for AD have persisted for over 20 years, during which numerous effective biomolecules for diagnosis, prognosis assessment, and even disease prediction have been identified [8,9]. The combined use of multiple CSF biomarkers is expected to significantly improve the accuracy and sensitivity of early diagnosis. However, given the invasive nature and potential side effects of CSF sampling, it is not feasible to screen for AD risk in asymptomatic populations using CSF samples [10]. Blood testing is widely used to assess the risk of arteriosclerosis, cerebrovascular disease, and cardiovascular disease in healthy, asymptomatic individuals [11,12]. At present, there is no effective, minimally invasive, and easily accessible blood biomarkers for AD diagnosis and risk prediction. A large number of blood samples from middle-aged and elderly healthy can be collected in regular physical examinations. Therefore, blood samples have greater advantages in developing biomarkers for AD in contrast to CSF [13]. Over the past 15 years, extensive research has explored blood biomarkers for the diagnosis and prognosis of AD, with some candidates becoming promising minimally invasive indicators. For example, multiple studies have indicated that the reduced level of Aβ and the elevated level of p-Tau in blood may reflect the deposition of amyloid proteins and the formation of neurofibrillary tangles in the early stages of AD [14,15]. Although further research is needed to confirm the changes in blood proteins and other indicators in AD patients, substantial evidences support blood biomarkers as the next-generation tools for AD diagnosis [16]. Biosensors are highly sensitive, simple, rapid, and cost-effective analytical tools widely used in disease monitoring, drug development, and target detection. Up to now, many reviews have provided professional insights on detection of AD biomarkers [17,18,19,20,21], but only a few works focus on the detection of multiple blood proteins. In addition, most of them only discussed the achievement of electrochemical biosensors in certain aspects, or missed the progress of photochemical techniques and transistors for biomarker detection. In this review, blood-derived biomarkers for AD diagnosis are briefly summarized, including the core pathological proteins of Aβ and Tau, as well as other neurodegeneration and metabolism-related protein biomarkers. Then, the methods and principles of various electrochemical and electrical techniques used for determining protein biomarkers of AD in blood samples are comprehensively and systematically discussed, mainly including electrochemistry, electrochemiluminescence (ECL), photoelectrochemistry (PEC), quartz crystal microbalance (QCM), field-effect transistor (FET), and organic electrochemical transistor (OECT). We further provide the challenge during the transition from research to clinical application, and offer future perspectives on emerging techniques.

## 2. Blood Protein Biomarkers for AD

Aβ deposited in cerebral senile plaques is mainly composed of 40~43 amino acids. Among kinds of Aβ monomers, Aβ40 and Aβ42 are the main products produced by the sequential hydrolysis of amyloid precursor protein (APP). Normally, APP is cleaved into non-amyloid fragments by α-secretase, while Aβ40 and Aβ42 are produced by the sequential hydrolysis of APP under the enzymolysis of β-secretase and γ-secretase. Longer Aβ molecules, especially Aβ40 and Aβ42, are highly prone to aggregation and premature deposition in the brain. Among various Aβ aggregates, Aβ oligomer (AβO) shows the greatest toxicity to neurons. Therefore, Aβ40, Aβ42 and AβO are considered as the most commonly used indicators for diagnosing symptomatic or early-stage AD [22]. However, studies on plasma concentrations of Aβ40 and Aβ42 in AD patients yielded inconsistent and sometimes contradictory results. Some independent studies suggested that a lower plasma Aβ42/Aβ40 ratio in non-demented elderly individuals is associated with more severe cognitive decline. Patients with AD or mild cognitive impairment accompanied by brain amyloid deposition have significantly lower plasma levels of Aβ42 compared to cognitively normal individuals. Subsequent studies have confirmed that a decrease in the Aβ42/Aβ40 ratio is positively correlated with cerebral amyloid burden. The discrepancy may arise from the differences in clinical stage or inclusion of patients with other dementias. The unstable structure of Aβ40 and Aβ42 can undergo changes within a few hours, which imposes high technical requirements and necessitates validation through independent studies. For this view, Nakamura et al. demonstrated the measurement of high-performance plasma Aβ biomarkers by immunoprecipitation coupled with mass spectrometry, enabling broader clinical access and efficient population screening of AD [23].

Tau proteins play a crucial role in maintaining microtubule stability in central nervous system neurons. Human brain Tau proteins in neuronal axons consist of six soluble isoforms with multiple phosphorylation sites [18,24]. Hyperphosphorylated p-Tau can detach from microtubules to form insoluble aggregates known as neurofibrillary tangles in neurons. The total Tau and p-Tau in CSF are important indicators of neurodegenerative diseases. An elevated level of total Tau is a hallmark of neuronal damage, including AD, Creutzfeldt Jakob disease, Lewy body dementia, and frontotemporal dementia [14]. Compared with other neurodegenerative diseases, the levels of p-Tau in the CSF of AD patients are significantly elevated, reflecting the excessive phosphorylation of Tau and the formation of neurofibrillary tangles [14]. Due to the invasiveness and high cost of CSF testing, blood Tau proteins have become potential biomarkers for diagnosing AD. Extensive research has quantified the contents of different Tau isomers in the plasma of AD patients, mild cognitive impairment patients, and healthy control groups. It was found that the levels of plasma Tau in AD patients are significantly higher than that in the control groups or patients with mild cognitive impairment [25]. There is no difference between patients with mild cognitive impairment progressing to AD and those who remained stable. The analysis of two large cohorts suggested that the concentration of plasma Tau partially reflected AD pathology. Although the correlation is weak, common features were observed between AD patients and age-matched control groups, making plasma Tau a challenging individual biomarker for AD. Recently, elevated levels of p-Tau proteins such as p-Tau181, p-Tau217, and p-Tau231 have been found in the plasma of AD patients, making them become effective biomarkers for distinguishing AD dementia from other diseases [9,14]. Typically, Aarsland et al. determined p-Tau217 in 11,486 plasma samples from the individuals aged 57 and above for neuropathological changes [26], and found a higher prevalence of AD dementia in older individuals and a lower prevalence of preclinical AD in younger groups than previously estimated. It is worth noting that in recent years, the detection of blood protein biomarkers related to AD has shifted from research validation to clinical implementation. In 2025, the US Food and Drug Administration approved two blood tests, including Lumipulse G p-Tau217/Aβ42 plasma ratio and Roche Elecsys p-Tau181 plasma test, as auxiliary tools for AD diagnosis in adult patients [27,28]. Both of these detection methods are highly consistent with CSF analysis and PET imaging. The regulatory approval of these commercial platforms has validated the clinical utility of p-Tau181 and p-Tau217 as AD-related blood biomarkers.

Neurofilaments are intermediate silk proteins that form the structural components of neuronal axons. In the central nervous system, neurofilaments include four subunits: light chain neurofilament (NFL), medium chain neurofilament, heavy chain neurofilament, and α-reticulin. The release of neurofilament into CSF and blood circulation after neuronal injury has been widely studied in neurodegenerative diseases. The levels of NFL were significantly elevated in CSF and pasma of patients with frontotemporal dementia and late-onset AD [29,30]. For example, it was suggested that high concentrations of NFL in CSF can distinguish AD patients from healthy individuals, although elevated levels of NFL have also been observed in other neurodegenerative diseases [31]. Zetterberg and co-workers suggested that higher concentrations of NFL were associated with more severe cognitive decline and greater brain atrophy in patients with AD and mild cognitive impairment [32]. These studies suggested that NFL is a hallmark of AD progression, but it is a universal biomarker for neurodegenerative diseases rather than AD only.

In addition to biomolecules related to the pathogenesis of different neurodegenerative diseases, changes in the levels of other plasma proteins such as glial fibrillary acidic protein (GFAP) have also been widely studied as the biomarker of AD [12,33]. In addition, Hye and co-workers found 10 plasma proteins closely associated with the transition from mild cognitive impairment to AD, which may be caused by AD-induced neurodegenerative or systemic diseases [34]. Ray et al. identified 18 plasma signaling proteins for distinguishing AD or mild cognitive impairment patients from healthy controls with an accuracy rate of nearly 90% [35]. Leifert et al. identified a group of plasma proteins for distinguishing AD patients from healthy individuals with excellent sensitivity and specificity [36]. However, the low abundance of these proteins and the high detection cost hinder the systematic validation of their expression patterns in AD.

## 3. Electrochemical Biosensors

Electrochemical biosensors have advantages such as fast response speed, strong real-time monitoring capability, low cost, easy miniaturization, and intelligent detection function. Recognition element is a key component for the detection of target analytes, which can determine the specificity and sensitivity of biosensors. The generally used recognition elements include natural and synthetic receptors such as antibodies, DNA/RNA, peptides, enzymes, and molecular imprinting polymers (MIPs). According to the type of recognition elements, electrochemical biosensors can be divided into immunosensors, aptasensors, enzyme sensors, MIP sensors, and other recognition-based sensors. They typically involve the conversion of biological recognition events into electrical signals, including current, potential, or impedance. Among them, voltammetry and electrochemical impedance spectroscopy (EIS) are the most frequently employed techniques for biomarker detection due to their high sensitivity and excellent ability to monitor interface changes during target binding. Usually, converting biological recognition events into electrical signals requires electroactive substances in the systems. The common electroactive substances include signaling reporters labeled on antibodies, antigens, and DNA, redox mediators attached on electrode surface or distributed in the electrolyte solution, as well as electroactive products generated during enzymatic reactions.

Among kinds of electrochemical biosensors, immunosensors are a type of sensing techniques that employ antigens or antibodies as the biological recognition elements based on the highly specific antigen–antibody interaction. According to the differences in detection mechanisms, immunosensors can be classified into two categories: direct “antibody–antigen” detection and sandwich “antibody–antigen–antibody” detection. Electrochemical aptasensors with aptamers such as DNA, RNA, and peptides as recognition elements can specifically recognize target molecules by binding to their spatial conformation, secondary structure, or tertiary structure. Compared with natural antibodies, aptamers known as “chemical antibodies” have the advantages of low molecular weight, low immunogenicity, as well as easy of synthesis and modification. The currently reported electrochemical aptasensors for the detection of AD-related blood protein biomarkers can be divided into three categories. The first category is based on the direct aptamer–target interaction. The binding can cause a change in the impedance on the electrode surface, affecting the electron transfer of redox mediators in the solution. Additionally, the target-induced conformational change of aptamer can alter the electron transfer of redox tag labeled on the aptamer, ultimately leading to a measurable change in the current. The second category relies on the competitive binding between the target and the DNA/RNA sequence that is partially complementary to the aptamer. The complementary sequence released in the solution or at the interface will cause a change in the electrochemical signal and even serve as an initiator to trigger a DNA-based signal amplification for further improving the detection sensitivity. The third category is similar to the sandwich detection mode of “antibody–antigen–antibody”, which employs one or a pair of aptamers instead of antibodies for target capture and recognition. In addition, one of the recognition elements in a pair of antibodies or aptamers can be replaced by another recognition element such as peptide or MIP to form a sandwich structure.

### 3.1. Direct Detection

In the direct detection mode, recognition elements such as antibodies and aptamers are usually immobilized on the electrode surface through physical or chemical adsorption, embedding, covalent crosslinking, or self-assembly. When the recognition element binds to the target analyte, the electron transfer between the redox signaling molecules in the solution or on the sensor interface and the electrode will be hindered, resulting in a significant change in the electrochemical signal and enabling sensitive and specific detection of target through a concentration-response relationship. Nanomaterials have attracted widespread attention due to their small size, quantum/confinement effects, large specific surface area, and high reactivity. They are frequently used as the electrode modifiers to facilitate the immobilization of biological recognition elements and increase the effective surface area and electrical conductivity, thus enhancing the sensor performances such as reduced background current, improved signal-to-noise ratio, and accelerated electron transfer. The conductivity, stability, and clinical applicability of different electrode materials and their advantages and limitations are shown in Table 1.

**Figure 1:**
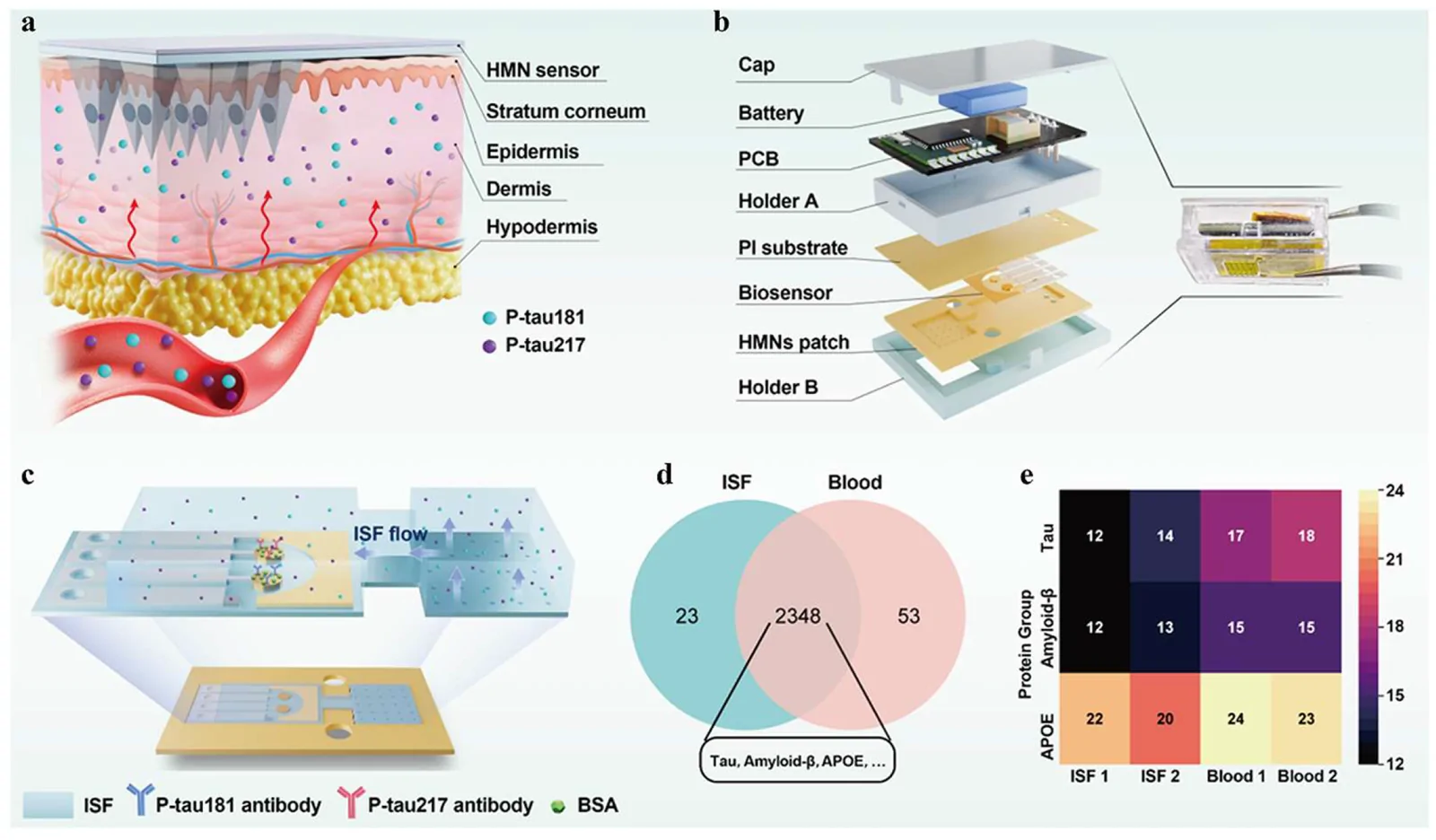
Schematic illustration of the integrated wearable electrochemical sensor for detecting p-Tau181 and p-Tau217 in an ISF. (**a**) ISF collection by microneedles. (**b**) The integrated wearable electrochemical sensing patch. (**c**) ISF flow within the HMN patch. (**d**) Analysis of protein species in mouse ISF and blood. (**e**) Heatmap of the relative concentrations of AD biomarkers (Tau, Aβ, and APOE) in mouse ISF and blood (control mice: ISF 1, blood 1; AD mice: ISF 2, blood 2). Reprinted with permission from ref. [54].

A variety of nanomaterials have been employed in electrochemical biosensors for the detection of AD biomarkers, including noble metal nanomaterials (e.g., gold and silver nanoparticles), semiconductor nanomaterials, carbon nanomaterials (e.g., carbon nanotubes, graphene oxide, carbon spheres), and metal-organic frameworks (MOFs) [37,38,39]. The performances of nanomaterials-modified sensing electrodes for immunosensing and aptasensing of Aβ and Tau proteins have been addressed in several previous reviews [40,41,42,43,44]. This section only showed and discussed several representative examples for the assays of blood samples, as shown in Table 2, including immunosensors with the electrodes of antibody-modified self-assemble monolayers (SAMs) [45,46,47,48], metal nanomaterials [49,50,51,52,53,54,55], graphene [56,57,58], carbon nanotubes [59,60], and composite materials [61,62,63,64,65,66,67], and polymers [68,69,70], as well as aptasensors with the electrodes of aptamer-modified metal nanoparticles [71,72,73,74,75], and composites [76,77,78]. For the direct detection of blood protein biomarkers, a typical example is that Sun et al. developed an “all-in-one” wearable electrochemical patch to address the defects of traditional detection schemes, including invasive lumbar puncture, expensive PET scans, and blood sampling inconvenience (Figure 1) [54]. The work innovatively adopted interstitial fluid (ISF) as the detection medium and integrated microneedle sampling and electrochemical sensing into one wearable device to realize painless, real-time in situ multi-index detection of p-Tau. The device consists of two core modules: biocompatible 3D printed hollow microneedle arrays for extracting ISF via capillary action, and dual working electrodes modified with vertical graphene-gold composite (VG@Au) to form a three-electrode electrochemical system. The specific binding of p-Tau181/p-Tau217 to their antibodies immobilized on the sensor surface reduced the electron transfer efficiency and decreased the differential pulse voltammetry current. The signal variation positively correlated with biomarker concentration for quantitative readout. The method achieved detection limits of 0.058 pg/mL for p-Tau181 and 0.079 pg/mL for p-Tau217, with a wide linear range, strong anti-interference against common biological interferents, and stable signal output within 14 days of storage. This integrated biosensor exhibited high selectivity toward p-Tau181 and p-Tau217 in the presence of common biological interferents, such as ascorbic acid, sucrose, glucose, and human serum albumin (HSA). Equipped with Bluetooth circuit, the patch could transmit the testing data to mobile terminals wirelessly. In animal sample verification, the wearable patch was applied to collect ISF from AD model mice and normal control mice. It was found that AD ISF showed significantly higher levels of p-Tau181 and p-Tau217 than the control groups. This integrated wearable patch provided a portable, minimally invasive tool for long-term early screening and therapeutic monitoring of AD.

**Figure 2:**
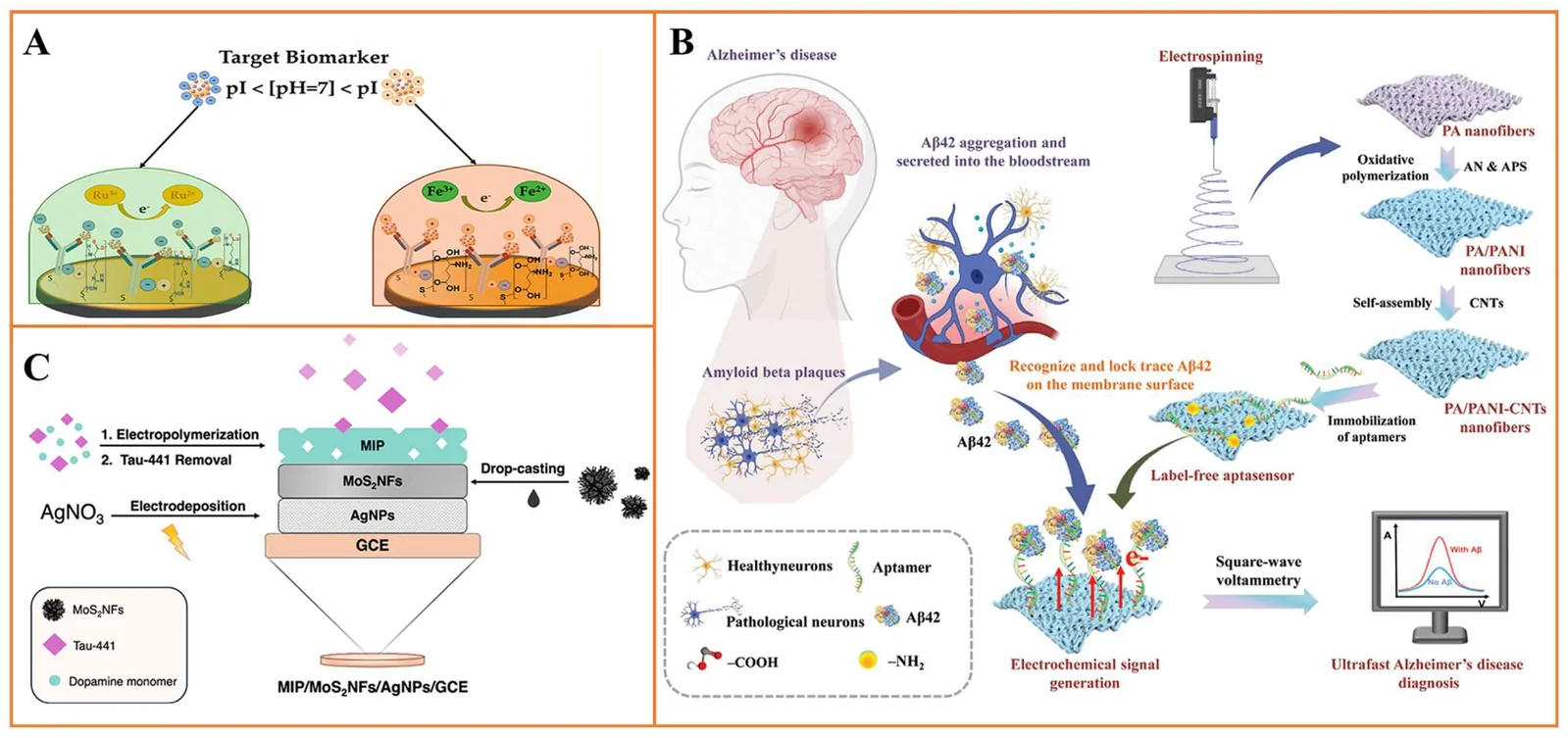
(**A**) Schematic illustration of neutral charged immunosensor for protein-based biomarker analysis. Reprinted with permission from ref. [79]. (**B**) Schematic illustration of detection mechanism of the PA/PANI-CNTs nanofiber-based A*β*42 aptasensor for ultrafast AD diagnosis. Reprinted with permission from ref. [80]. (**C**) Schematic illustration of the MIP nanosensor for Tau-441 detection. Reprinted with permission from ref. [84].

SAMs are the generally used interfaces for the immobilization of antibodies on precious metal electrodes for direct immunoassays of protein biomarkers. However, the formation and activation of SAMs for cross-linking of antibodies are complex and time-consuming, making this method undesirable for the manufacture of disposable biosensors for routine clinical application. In addition, the uneven surface layer of SAMs may lead to non-specific binding and alignment issues. For this consideration, Dai et al. constructed a neutral charged immunosensor (NCI) to solve the defects of traditional electrochemical immunosensors, such as complex surface modification, severe background electrostatic interference, and only applicability to negatively charged proteins (Figure 2A) [79]. The design strategy introduced the oppositely charged polyamino acid neutralizers to offset the intrinsic surface charge of immobilized antibodies, eliminating background electrical signals and only retaining the electrostatic response generated by target proteins. The platform could simultaneously detect both acidic and alkaline isoelectric point protein biomarkers. For acidic pI targets like HE4, polyarginine was used as the positive neutralizer under alkaline buffer with cationic ruthenium redox probes. For alkaline pI targets such as total Tau, polyglutamic acid was used as negative neutralizer in acidic environment matched with anionic ferrocyanide probes. After the specific antigen-antibody binding, the electrostatic attraction between target proteins and redox probes caused the change of electrode diffusion current, which could be monitored by differential pulse voltammetry. This biosensor featured ultrahigh picomolar sensitivity, with the detection limits of 2.5 pM for HE4 and 0.968 pM for Tau, one order of magnitude lower than non-neutralized electrodes. The whole preparation process took less than 2 h and single test could be performed within 30 min. In human serum actual samples, the platform exhibited strong anti-interference against homologous disease biomarkers including CA-125, Aβ42, and TDP-43, with stable linear calibration curves and low relative standard deviations. This simple, universal electrochemical sensing strategy provided a low-cost candidate for clinical point-of-care simultaneous detection of neurodegenerative and tumor protein biomarkers.

Polymer nanocomposites can enhance the performance of biosensors by combining the advantages of two or more types of polymers, such as improved mechanical stability, high electrical conductivity, and good biocompatibility. The nanocomposites can promote the adsorption and detection of biomarkers, provide stable electrochemical signals, and improve detection sensitivity and selectivity by adjusting polymer composition and structure. Polyaniline is an intrinsically conductive polymer. When combined with carbon nanotubes, it can further improve the overall conductivity of the composites, thereby increasing the detection efficiency of biosensors. Liu et al. proposed the detection of blood Aβ42 using polyamide/polyaniline-carbon nanotube (PA/PANI-CNT)-modified electrodes (Figure 2B) [80]. A freestanding nanofiber membrane loaded with DNA probes was prepared by electrospinning, in which PA/PANI-CNT nanofiber was used as a self-supporting scaffold for aptamer immobilization. The label-free aptasensor enabled accurate and fast detection of Aβ42 within 4 min, and had excellent selectivity even in the presence of other proteins at a 100-fold higher concentration. The method showed excellent selectivity, low detection limit, wide linear range, good reproducibility, and long-term storage stability for the assays of Aβ42 in human serum samples.

MIP offers promising an alternative for antibody to recognize protein biomarkers due to its biomimetic recognition, cost-effectiveness, and ease of preparation. Recently, MIP-based electrochemical biosensors have been developed for the detection of AD-related blood protein biomarkers [81,82,83,84,85,86,87,88]. For instance, Turan et al. designed a MIP-based electrochemical biosensor for rapid and selective detection of Tau-441 (Figure 2C) [84]. In this work, silver nanoparticles (AgNPs) were electrodeposited on the glassy carbon electrode coated with 3D molybdenum disulfide (MoS_2_) nanoflowers and dopamine was electropolymerized in the presence of Tau-441 to form an imprinting layer. The biosensor exhibited a wide linear range (1.93 fM–100 pM), a low detection limit (0.59 fM), as well as good recovery and selectivity for the assays of Tau-441 in human serums and artificial CSF, and showed excellent reproducibility with 68.47% of initial response after 12 days. In addition, Deshpande’s group developed a low-cost point-of-care electrochemical biosensor for direct detection of p-Tau181 in undiluted plasma using polyphenol red (pPhR) MIP-modified highly porous gold (HPG) electrode [85]. HPG provided a large electroactive surface area and antifouling property, while electropolymerized pPhR acted as an internal redox reporter to eliminate the requirement of additional exogenous redox probes. Integrated homemade portable potentiostat and machine learning network realized automatic signal classification and regression analysis, achieving 100% diagnostic accuracy and low detection limit (980 fg/mL) without false positives or negatives. This modular artificial intelligence-aided platform complies with the decentralized diagnosis standard of World Health Organization and can be extended to detect multiplex neurodegenerative biomarkers.

**Figure 3:**
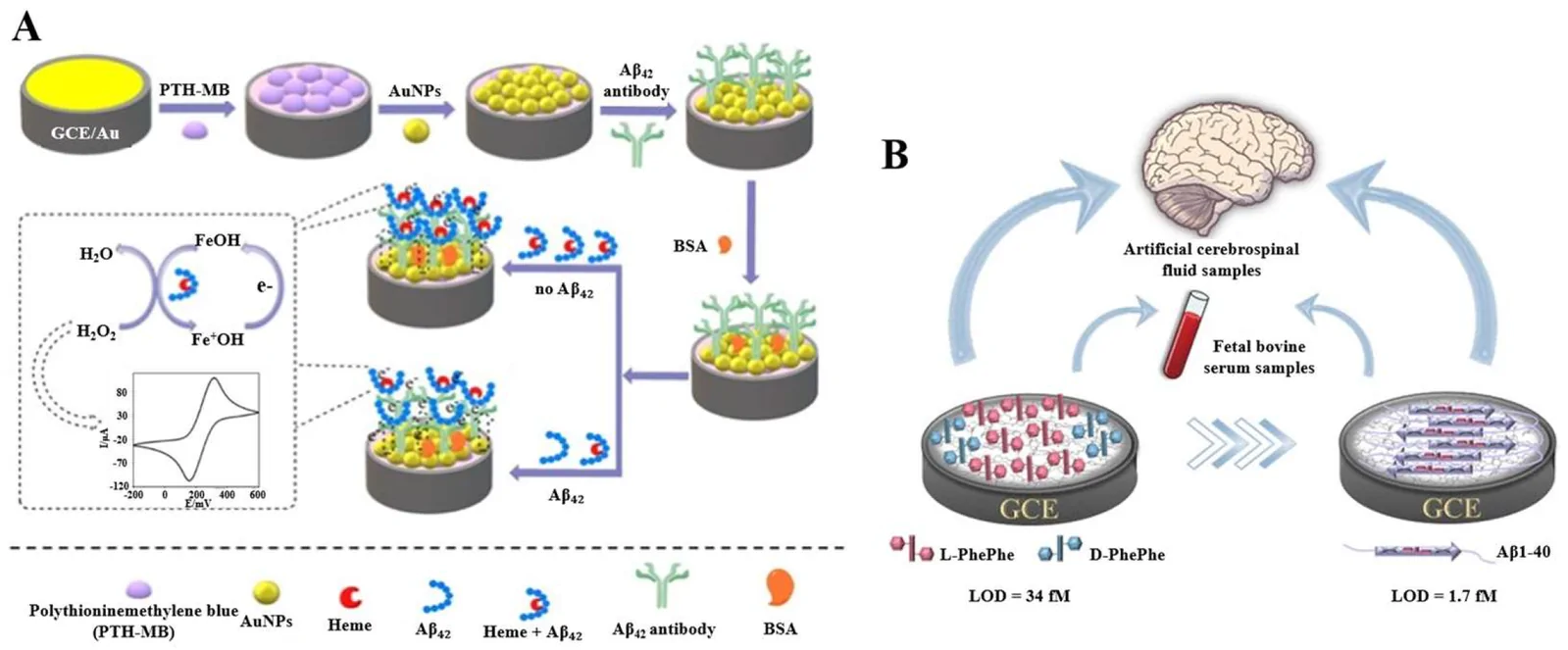
(**A**) Schematic illustration of electrochemical immunosensor for Aβ42 detection. Reprinted with permission from ref. [89]. (**B**) Schematic illustration of electrochemical Aβ biosensor with homochiral MOFs binding to the L-diphenylalanine targeting sites. Reprinted with permission from ref. [90].

Aβ can coordinate with metal ions or metal-containing molecules/materials to generate measurable electrochemical signals. It has been reported that Aβ can bind with copper ion or iron-containing heme to form peroxidase-like enzyme with good catalytic activity. Ding et al. developed an electrochemical method for Aβ42 detection based on the peroxidase-like activity of Aβ42–heme, where the captured Aβ42–heme complex catalyzed the electron transfer between hydrogen peroxide and ferrocenemethanol (FcOH) (Figure 3A) [89]. Free Aβ42 in the sample could compete with the Aβ42–heme complex to bind with the antibody immobilized on the sensor surface, thereby reducing the electrochemical signal. This biosensor showed a detection limit of 17.3 pM (78.09 pg/mL) for Aβ42 detection in phosphate-buffered saline, 25.2 pM (113.75 pg/mL) in serum, and 23.8 pM (107.43 pg/mL) in saliva. This immunosensor also presented good selectivity toward Aβ42 against Aβ40, HSA, and lysozyme.

All of the aforementioned bioreceptor-based biosensors can achieve specific recognition and sensitive detection. However, the strict requirement and high cost for the preparation and storage of bioreceptors will hinder their clinical translation. In this aspect, novel electrode materials that can simultaneously perform molecular recognition and signal transduction will be able to promote the practical application of electrochemical biosensors. Li et al. developed a bioreceptor-free electrochemical biosensor for Aβ detection using a chiral MOF ([Zn_4_(BPIle)_4_(bpta)_2_]·3DMF·3H_2_O) that can specifically target Aβ with the Phe-Phe motif (Figure 3B) [90]. The target recognition relied on the framework structure, hydrophobicity, and chirality of MOF rather than the specific biometric response. The strong π–π interaction between MOF and aromatic side chain led to a low detection limit of 1.7 pM (7.36 pg/mL) for effective detection of Aβ spiked in serum and artificial CSF with 96% recovery. However, the specificity of this method for other amyloid peptides and proteins including Phe-Phe motifs has not been investigated.

**Figure 4:**
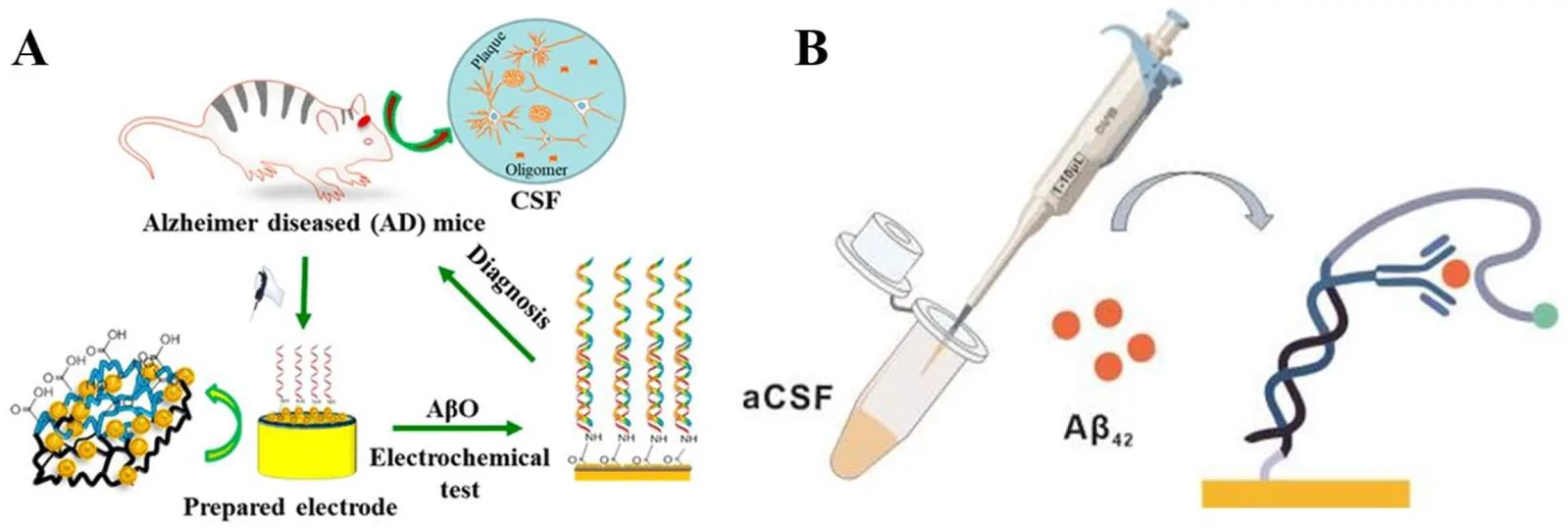
(**A**) Schematic illustration of AuNPs−PEDOT−PTAA/PrP^C^ sensor for AβO detection. Reprinted with permission from ref. [96]. (**B**) Schematic illustration of Ab-Apt chimeric molecular pendulum for Aβ42 analysis. Reprinted with permission from ref. [101].

Besides nucleic acid aptamers, peptides with high affinity and specificity can be screened from synthetic peptide libraries through in vitro selection techniques to serve as the bioreceptors. With peptides as the specific recognition elements, various biosensors have been designed for the assays of AD-related protein biomarkers [91,92,93,94,95,96]. For example, based on the studies that AβO can bind with the N-terminal residues of cellular prion protein (PrP^C^) to mediate the neuronal toxicity of Aβ aggregates [97,98], Qin et al. constructed a label-free electrochemical impedance biosensor for AβO detection by employing a conductive AuNPs-modified poly(3,4-ethylene dioxythiophene)−poly(thiophene-3-acetic acid) (PEDOT−PTAA) composite as the electrode substrate (Figure 4A) [96]. The AuNPs-embedded PEDOT interlayers greatly enhanced the electrode conductivity and surface area, while poly(thiophene-3-acetic acid) provided abundant carboxyl sites for PrP^C^ immobilization. The specific binding between PrP^C^ and AβO hindered interfacial electron transfer and raised charge transfer resistance, establishing a quantitative correlation between impedance variation and AβO concentration. The sensor achieved a wide linear range of 10^–8^~10^4^ nM and a sub-femtomolar detection limit, and could selectively distinguish AβO from fibrils. The in vitro experiments of AD model mice validated the application of this biosensor for early minimally invasive diagnosis of AD by monitoring the change of AβO concentration. However, immobilization of aptamers on the electrode surface through Au-S bonding remains a challenge due to the biofouling and biothiol interference. For this consideration, Cheng et al. designed an antifouling electrochemical biosensor for the determination of Aβ42 using palladium nanoparticles (PdNPs)-modified electrode to immobilize a thiolated multifunctional peptide (SFP: CEKEKEKEKERGTFEGKF) through Pd-S bonding [99]. It was found that the Pd-S bond exhibited a higher binding energy, shorter bond length, and enhanced electron transfer compared to the Au-S bond. The peptide-based biosensor showed a linear range of 0.1 pg/mL~1 μg/mL and a detection limit of 0.038 pg/mL with consistent performance in buffer and serum. Clinical validation for serum samples exhibited good agreement with enzyme-linked immunosorbent assay (ELISA), indicating its accuracy and potential for AD diagnosis.

Most of the above discussed methods employ a single recognition element as the molecular switch for target detection, which may limit their adjustability and regulatory range [100,101]. Very recently, Liu et al. developed a reagent-free molecular switch electrochemical biosensor with antibody-aptamer dual-recognition architecture to overcome the limited recognition flexibility of traditional single-recognition sensing platforms (Figure 4B) [101]. The dual-recognition probe can simultaneously bind to two independent epitopes on the target protein, triggering large-scale conformational rearrangement of the DNA switch and greatly amplifying the intrinsic electrical signal in contrast to dual-aptamer counterparts. Low-temperature freeze surface self-assembly treatment accelerated uniform probe immobilization and further reduced the detection limit. By adjusting the applied voltage and holding time, the sensing electrode can be fully regenerated within 30 s for repeated use. The biosensor displayed outstanding anti-interference capacity and achieved sensitive quantitation of target proteins such as HSA and Aβ in serum and artificial CSF samples, showing broad application potential for continuous monitoring of disease-related protein biomarkers.

**Figure 5:**
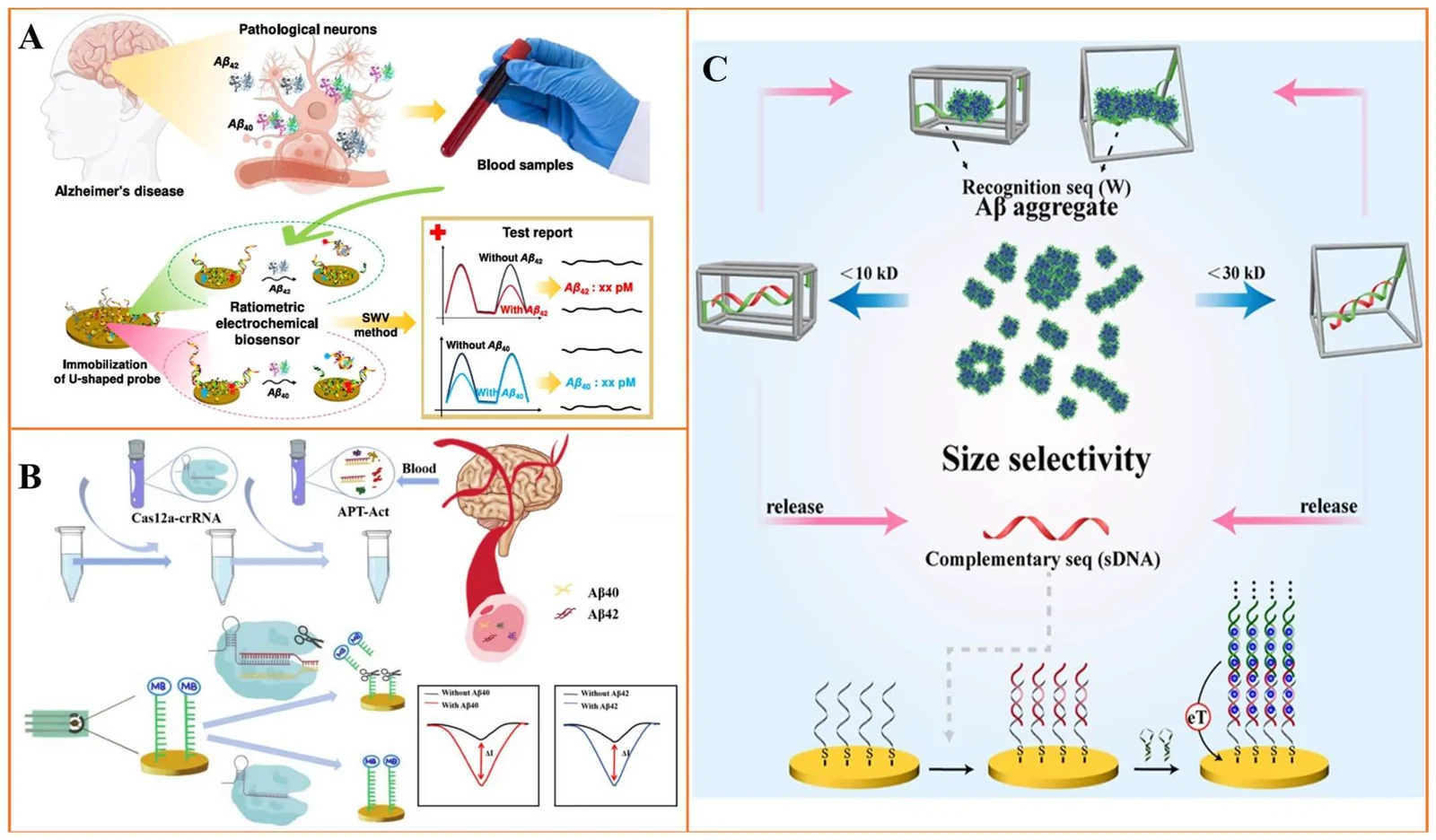
(**A**) Schematic illustration of Au nanoelectrodes and mechanism for Aβ40 and Aβ42 detection. Reprinted with permission from ref. [103]. (**B**) Schematic illustration of the Cas12a/crRNA/APT-Act system for for Aβ40 and Aβ42 detection. Reprinted with permission from ref. [104]. (**C**) Schematic illustration of the DNA nanocage-assisted recognition and determination platform for LSAβO. Reprinted with permission from ref. [107].

Electrochemical aptasensors can combine sequence-specific molecular recognition with redox reporters capable of generating measurable electrical signals. The electroactive molecules such as ferrocene (Fc), methylene blue (MB), and anthraquinone (AQ) can be placed at the 5′ end, 3′ end, or internal position of aptamers. The conformational change of aptamer can alter the electron transfer of redox tag labeled on the aptamer, ultimately leading to a measurable change in the current. Based on this principle, blood protein biomarkers have been determined for AD diagnosis [102]. For example, Tong et al. fabricated a poly-A-based ratiometric electrochemical aptasensor with signal displacement probes for the accurate and non-invasive detection of AD biomarkers, particularly Aβ40 and Aβ42 (Figure 5A) [103]. To improve the detection sensitivity and stability, AuNPs were electrochemically deposited on the electrode surface for the immobilization of aptamers, offering a significantly increased surface area and promoting electron transfer. The sensing principle relied on the self-assembled poly-A-mediated U-shaped DNA structure containing an aptamer region for target recognition. The poly-A sequence enabled robust, label-free anchoring of DNA probes on the surface of AuNPs, eliminating the complex thiol-based modification typically required in conventional systems. This approach simplified sensor fabrication while maintaining high binding affinity and surface stability. Upon specific aptamer-target interaction, the target Aβ peptide displaced the signal probe from the electrode surface, leaving the reference probe unaffected. Response calibration using the reference probe could normalize signal changes and minimize fluctuations caused by environmental or sample variations. This method significantly enhanced reliability and anti-interference in complex biological matrices. It achieved detection limits of 21 pM for Aβ40 and 59 pM for Aβ42, with high recoveries of 96.7–102.2% in spiked serum. A significant advantage of this sensor architecture is the direct quantification of the Aβ42/Aβ40 ratio, an effective indicator of AD progression, using appropriate reference probes. In addition, Yuan et al. reported an aptamer-CRISPR system using disposable screen-printed electrode as the detection platform (Figure 5B) [104]. The aptamer activator system could specifically bind with the target protein and release activator DNA (actDNA), activating the trans-cleavage ability of Cas12a. Non-specific cleavage of MB-labeled single-stranded DNA reporter probe caused a decrease in peak current, which is indicative of the presence of target analyte. The detection limits of this method for Aβ40 and Aβ42 were as low as 1.1 pg/mL and 0.8 pg/mL, respectively. The disposable electrochemical aptasensor is simple and particularly suitable for resource-limited settings.

Target-induced release of nucleic acid sequences in the solution or at the interface as initiators to trigger signal amplification is rapidly becoming an industry standard for disease diagnosis by integration with the techniques of polymerase chain reaction (PCR), rolling circle amplification (RCA), catalytic hairpin assembly (CHA), loop-mediated isothermal amplification (LAMP), ligase chain reaction (LCR), hybridization chain reaction (HCR), strand displacement amplification (SDA) and others [105,106]. The method has also been used to diagnose AD by monitoring the concentration changes of blood protein biomarkers [104,107,108,109,110,111,112,113,114]. For example, Xiang’s group designed a series of DNA nanocages with different sizes and employed them to screen and detect specific large soluble AβO (LSAβO) based on the size-dependent effect (Figure 5C) [107]. Two DNA nanocages matching LSAβO10kD and LSAβO 30kD were found and functionally modified by introducing aptamers and complementary sequences into their cavities. The presence of target could trigger the subsequent HCR to generate an amplified electrochemical signal. The method showed linear ranges of 10−150 pM for LSAβO10kD and 15−150 pM for LSAβO30kD and has been used to determine LSAβO in serum for the first time.

**Table 2:** Performance of direct electrochemical methods for the detection of AD-related blood protein biomarkers.

Method	Electrode Material	Biomarker	Linear Range	Detection Limit	Ref.
Immunoassay	SAM	p-Tau	0.1–3 × 10^3^ pM	0.05 pM	[45]
	SAM	Tau	1–10^2^ ng/mL	1 ng/mL	[46]
	SAM	Aβ42	10^−3^–10^3^ ng/mL	500 pg/mL	[47]
	protein G	Tau	0.01–10^4^ pM	0.03 pM	[48]
	AuNPs	Tau-441	50–10^7^ fg/mL	16 fg/mL	[49]
	AuNPs	GFAP, p-Tau181	10–10^3^ pg/mL, 0.1–1 pg/mL	9.95 pg/mL, 0.044 pg/mL	[50]
	AuNDs	Tau, ApoE, Aβ	0.1–10^2^ pg/mL	59.1 fg/mL, 71.4 fg/mL, 8.6 fg/mL	[51]
	p(TB)/AuNPs/AuNSs	p-Tau181	1–10^5^ pg/mL	1.09 pg/mL	[52]
	LSG NDs	Aβ42	0.1−10^4^ pg/mL	84 fg/mL	[53]
	VG@Au	p-Tau181, p-Tau217	0.1–10^3^ pg/mL	0.058 pg/mL, 0.079 pg/mL	[54]
	VG@nAu	Aβ40, Aβ42, total Tau, p-Tau181	0.1–10^3^ pg/mL	0.072, 0.089, 0.071, 0.051 pg/mL	[55]
	GO/pPG	MBP, Tau	0.5–25 nM, 0.25–25 nM,	0.3 nM, 0.15 nM	[56]
	rGO/β-CD	p-Tau181	10^−3^–10^3^ pg/mL	0.92 fg/mL	[57]
	Cu^2+^/PTSA@rGO	Tau-441	0.08–80 pM	75 fM	[58]
	TpPa@CNT	Tau	10^−3^–10^4^ ng/mL	0.33 pg/mL	[59]
	PEDOT-PSS/MWCNT	Tau-441	10–5 × 10^7^ pg/mL	7.4 pg/mL	[60]
	rGO/AuNP	Tau-441	1–500 pg/mL	91 ng/mL	[62]
	MWCNT/dPEDOT	p-Tau217	0.1–10^5^ pg/mL	0.1 pg/mL	[63]
	MWCNT/Pt	p-Tau181	8.6–1.1 × 10^3^ pg/mL	0.24 pg/mL	[64]
	Co-MoS_2_/Au-rGO	AβO	10–1.1 × 10^5^ fM	6.87 fM	[65]
	Th-rGO-MWCNT	AβO	0.0443–4.43 × 10^2^ pM	10 fM	[66]
	IrNPs@COF	Aβ42	1–10^6^ μg/mL	0.65 pg/mL	[67]
	MHBN-APTES	Tau	0.5–50 pg/mL	0.39 pg/mL	[68]
	GPDMMS	Aβ42	1–100 pg/mL	0.37 pg/mL	[69]
	PDDA/G4	Tau	10^−2^–10^2^ ng/mL	1.31 pg/mL	[70]
Aptasensor	Apt-AuNPs	p-Tau231	10–10^7^ pg/mL	2.31 pg/mL	[71]
	Apt/AuNPs	AβO	10–10^8^ pg/mL	2.3 pg/mL	[72]
	Apt/AuNPs	AβO	1–10^4^ fM	0.5 fM	[73]
	Apt/AuNPs	p-Tau231	10–10^7^ pg/mL	2.31 pg/mL	[71]
	Apt/CFP/AuPt	AβO	0.5–10^4^ pg/mL	0.16 pg/mL	[74]
	Apt/VG@Au	Tau	0.1–10^3^ ng/mL	34 ng/mL	[75]
	Apt/Pt@ZIF-8	Tau	1–10^7^ fg/mL	1 fg/mL	[76]
	Apt/SnS_2_ NSs	AβO	10^−4^–10^3^ ng/mL	56.9 fg/mL	[77]
	Apt/PA/PANI-CNT	Aβ42	0.1–1.1 × 10^5^ pg/mL	30 fg/mL	[80]
	Apt/AuNPs/GO-MWCNT	Aβ	0.1–10^3^ ng/mL	88 fg/mL	[78]
MIP	AMP MIP	p-Tau-441	2.18–2.18 × 10^3^ pM	0.067 pM	[82]
	pPhR-pPy/MIP@hPG	p-Tau181	0–22.5 pg/mL	498 fg/mL	[83]
	pPhR MIP/HPG	p-Tau181	0.5–16 pg/mL	0.98 pg/mL	[85]
	MIP/MoS_2_/AgNPs	Tau-441	1.93–10^5^ fM	0.59 fM	[84]
	PTH-MB-ERG@MIP	Aβ42	0.12–10 μg/mL	18 pg/mL	[86]
	o-PDA/AuNPs	Tau-441	2–2 × 10^5^ pg/mL	1.51 fg/mL	[87]
Others	Peptide/AuNPs	Aβ42	0.3–500 fM	0.1 fM	[91]
	AuNPs−PEDOT−PTAA/PrP^C^	AβO	10^−8^–10^4^ nM	0.01 fM	[96]
	Peptide/PdNPs	Aβ42	0.1–10^6^ pg/mL	0.038 pg/mL	[99]
	Apt@Ab/AuNPs	Aβ40, Aβ42, total Tau, and p-Tau181	10–1000 pg/mL, 10–800 pg/mL, 10–500 pg/mL, 10–500 pg/mL	0.125 pg/mL, 0.089 pg/mL, 0.142 pg/mL, 0.176 pg/mL	[100]
	Ab-Apt	Aβ42	0.05–10^3^ nM	0.05 nM	[101]
	Apt/AuNPs	Aβ40, Aβ42	0.1–10 nM	59 and 21 pM	[103]

Abbreviation: SAM, self-assemble monolayer; AuNPs, gold nanoparticles; p(TB), poly(toluidine blue); AuNSs, gold nanostars; AuNDs, gold nanodendrites; LSG NDs, leaf-shaped gold nanodendrites; VG@Au, nanoAu-modified vertical graphene; GO, graphene oxide; pPG, amine functionalized 1st generation trimethylolpropane tris[poly(propyleneglycol)]; rGO, reduced graphene oxide; β-CD, β-cyclodextrin; PTSA, 1,3,6,8 pyrenetetrasulfonic acid tetrasodium salt; TpPa@CNT, amino-functionalized covalent organic frameworks and carboxylated carbon nanotubes; PEDOT:PSS/MWCNT, poly(3,4-ethylenedioxythiophene)-poly(styrene sulfonate)/carboxylated multi-walled carbon nanotube; Th, thionine; IrNPs@COF, iridium nanoparticle-loaded covalent organic framework; MHBN-APTES, magnetic hexagonal boron nitride-(3-Aminopropyl) triethoxysilane; GPDMMS, 3-glycidoxypropyldimethoxymethylsilane; PDDA, poly dimethyl diallyl ammonium chloride; G4, mucus-like guanosine-based hydrogel; Apt, aptamer; CFP/AuPt, carbon fiber paper with AuPt alloy nanoparticles; ZIF-8, zeolitic imidazolate framework-8; SnS_2_ NSs, tin disulfide nanosheets; PA, polyamide; PANI, polyaniline; CNT, carbon nanotube; AMP, 3-aminophenol; MIP, molecularly imprinted polymer; pPhR-pPy, polyphenol red and polypyrrole; hPG, highly porous gold; AgNPs, silver nanoparticles; PTH-MB-ERG and poly(thionine-methylene blue) and electrochemical reduction graphene oxide; o-PDA, electropolymerising phenylenediamine; PTAA, poly(thiophene-3-acetic acid); PdNPs, palladium nanoparticles; PrP^C^, cellular prion protein; Ab, antibody; GFAP, glial fibrillary acidic protein; ApoE, apolipoprotein E; MBP, myelin basic protein.

### 3.2. Sandwich-Type Detection

To further enhance the sensitivity and selectivity of electrochemical biosensors, researchers have proposed sandwich-type detection modes for AD diagnosis by determining the concentrations of protein biomarkers (Table 3). A typical sandwich-type biosensor consists of a primary antibody (Ab_1_) as the capture element, an antigen as the target analyte, and a secondary antibody (Ab_2_) modified with redox/catalytic label or nanomaterial as the signal readout element. Ab_1_ is immobilized on the sensor surface to specifically recognize and capture antigen, and subsequently, Ab_2_ is added to recognize the captured target. In this strategy, the catalytic label such as natural enzyme or synthetic nanozyme can catalyze the production of electrochemical substance or the direct electrooxidation of substrate with the help of a redox mediator [115,116,117,118,119]. Nanomaterials can be used as the carriers to load abundant electroactive probes [120,121,122,123], or as electrocatalysts to directly accelerate the electron transfer between the substrate and the sensor electrode [124,125,126,127,128,129,130]. Since the electrochemical signal originates from the signal label modified on Ab_2_, the attachment of numerous signaling tags on the sensor surface enables signal amplification by different strategies. Based on different signal transduction and amplification mechanisms, the sandwich-type detection method outperforms the direct detection mode in terms of sensitivity and specificity. Here are the representative examples for the determination of different AD-related protein biomarkers in blood samples.

**Figure 6:**
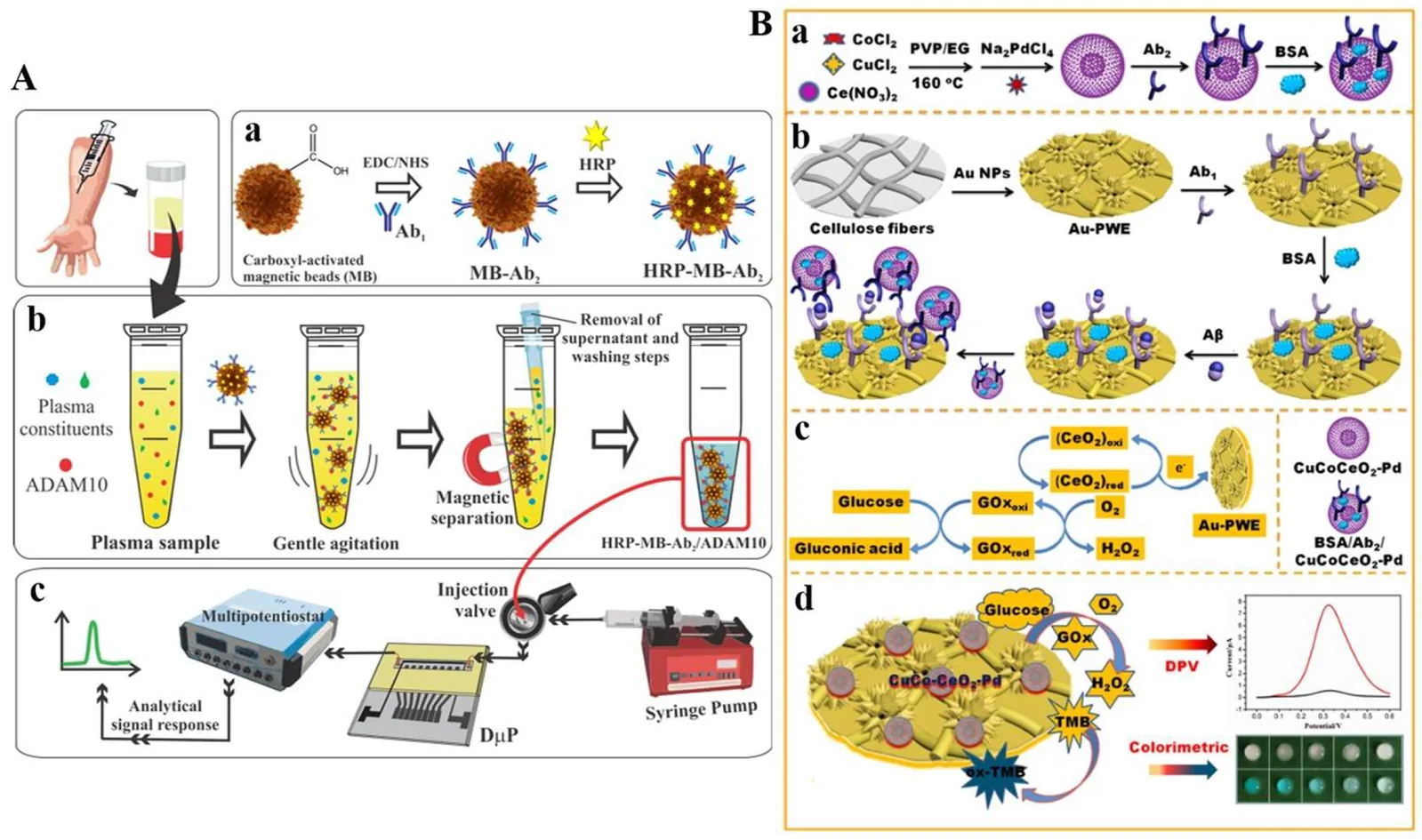
(**A**) Schematic illustration of immunomagnetic capture of ADAM10 in a plasma sample and the detection using DμP. (**a**) Conjugation of the MBs with HRP and Ab_2_. (**b**) ADAM10 immunomagnetic capture in the sample. (**c**) Setup of the microfluidic system and the transient current signal. Reprinted with permission from ref. [118]. (**B**) Schematic illustration of synthesis of CuCo-CeO_2_-Pd nanospheres and immobilization of Ab_2_ (**a**), construction procedure of oPAD (**b**), and differential pulse voltammetry-colorimetric dual-mode sensing of Aβ (**c**,**d**). Reprinted with permission from ref. [129].

A Disintegrin and Metalloprotease 10 (ADAM10), also known as α-secretase, is responsible for cleaving APP in the non-amyloidogenic pathway to produce soluble precursor proteins. Faria et al. developed a simple, low-loss, sensitive, and disposable microfluidic platform (DμP) for the early diagnosis of AD using ADAM10 as a blood biomarker (Figure 6A) [118]. This platform consisted of eight working electrodes that were coated with poly(diallyldimethylammonium chloride)/gold nanoparticles (PDDA/AuNP) for the immobilization of capture antibody Ab_1_. Magnetic nanoparticles (MNPs) conjugated with horseradish peroxidase (HRP) and Ab_2_ were used to collect ADAM10 from the samples. The HRP-MNP-Ab_2_/ADAM10 conjugate was then injected into the disposable microfluidic platform and captured by the surface-immobilized Ab_1_. The sandwich-type immunoassay could accurately detect ADAM10 in serum with a detection limit of 0.35 fg/mL. The subjects for ADAM10 detection included cognitively healthy individuals, patients with mild cognitive impairment, and AD patients at different disease stages. Receiver operating characteristic (ROC) curves showed that this platform could detect ADAM10 in human plasma with 72% sensitivity, 100% specificity, and an area under the curve (AUC) of 0.888. This method was used to accurately distinguish individuals with the sensitivity significantly better than that of the established ELISA method. In addition, Cui et al. built an origami paper-based analytical device (oPAD) with Cu/Co co-doped ceria-loaded palladium nanospheres as dual-signal amplifiers for electrochemical and colorimetric dual readout of Aβ (Figure 6B) [129]. Urchin-shaped AuNPs were in-situ grown on the cellulose paper to increase the immobilization sites for capture antibodies. The CuCo-CeO_2_-Pd composites exhibited high peroxidase-like electrocatalytic performance due to their abundant oxygen vacancies and high Ce^3+^/Ce^4+^ ratio. In the presence of glucose oxidase (GOx) and glucose, the captured composite catalyzed the generation of H_2_O_2_ to produce distinct differential pulse voltammetry currents and visible blue TMB indicators. The electrochemical chip achieved a detection limit of 0.05 pM and the naked-eye colorimetric mode reached a detection limit of 0.5 pM. The origami folding structure realized automatic washing and liquid switching, laying a foundation for low-cost portable point-of-care testing of AD.

**Figure 7:**
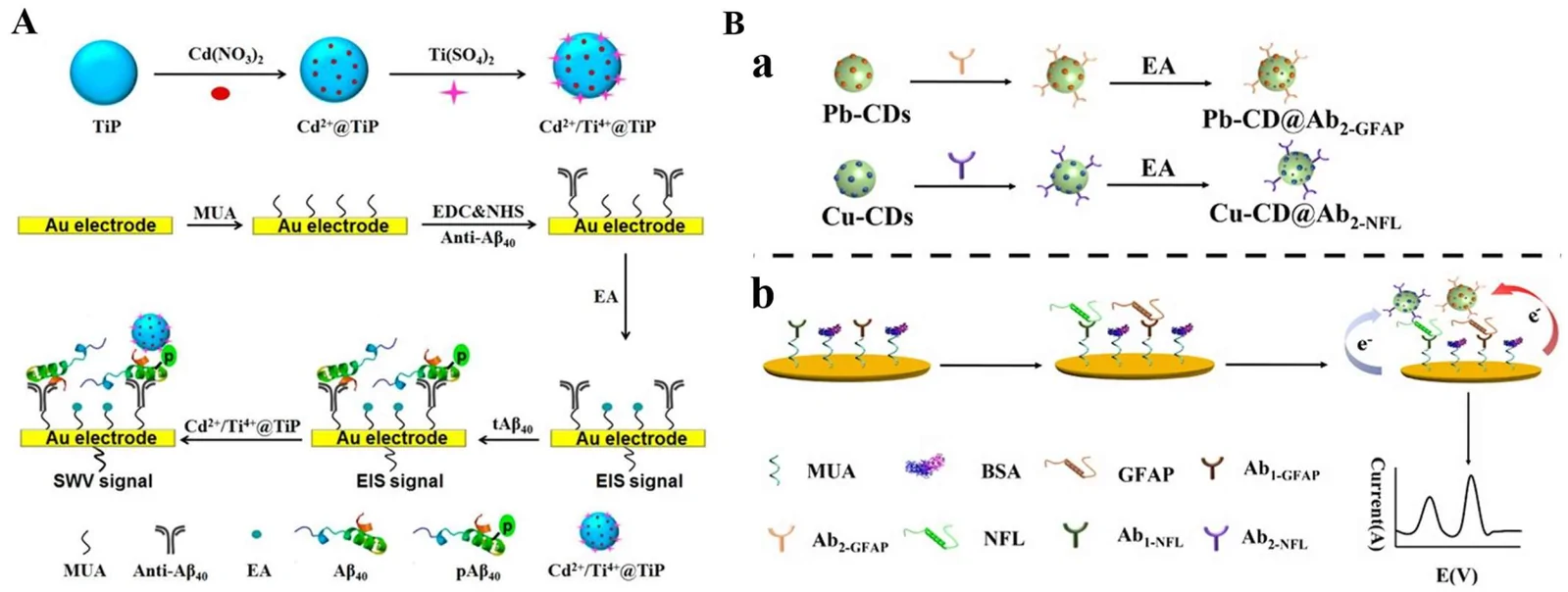
(**A**) Schematic illustration of preparation of Cd^2+^/Ti^4+^@TiP and construction of electrochemical biosensor. Reprinted with permission from ref. [133]. (**B**) Schematic illustration of the electrochemical quantification of GFAP and NFL. (**a**) Preparation of signal probes (Pb-CD@Ab_2-GFAP_ and Cu-CD@Ab_2-NFL_). (**b**) Simultaneous detection of two proteins. Reprinted with permission from ref. [134].

The relative content of phosphorylated Aβ40 (pAβ40) can serve as an effective auxiliary diagnostic index to improve clinical diagnostic accuracy since it shows significant statistical differences between AD patients and healthy individuals. Phosphate groups on the surface of phosphorylated proteins/peptides can be used as recognition sites. The specific interactions between metals or metal complexes and phosphates have attracted widespread attention for the separation and determination of phosphate-containing biomolecules [131,132]. Based on this fact, Yin et al. designed an electrochemical sensing strategy with a single antibody to synchronously quantify total Aβ40 (tAβ40) and pAβ40, addressing the limitations of multi-channel equipment and large concentration gap interference in traditional dual-probe detection (Figure 7A) [133]. Anti-Aβ40 antibody exhibited nearly identical binding affinity to both Aβ40 and pAβ40, enabling one-step co-immobilization of the two analytes on the gold electrode for tAβ40 quantification by electrochemical impedance technique. Titanium phosphate nanospheres functionalized with Cd^2+^/Ti^4+^ ions (Cd^2+^/Ti^4+^@TiP) could selectively recognize the phosphorylated site of pAβ40, generating an amplified square wave voltammetry signal. In addition, Zhang et al. developed an electrochemical sensing platform for the simultaneous detection of GFAP and NFL, two important proteins that maintain brain structure and function (Figure 7B) [134]. Carbon dots (CDs) doped with a large amount of Pb^2+^ or Cu^2+^ (Pb-CDs or Cu-CDs) were used as the probes to achieve dual-signal output. The detection limits were found to be 0.3 pg/mL for GFAP and 0.2 pg/mL for NFL. Serum sample analysis results showed that the concentration ratio of GFAP to NFL exhibited good specificity in distinguishing major depressive disorder, AD, and cerebral thrombosis. This work provided an inspiration for developing dual-signal sensing platforms, which is of great significance for early diagnosis of AD.

**Table 3:** Performance of sandwich-type electrochemical methods for the detection of AD-related blood protein biomarkers.

Signal Label	Biomarker	Linear Range	Detection Limit	Ref.
Antibody/HRP	p-Tau181	6.97–10^8^ fg/mL	1.91 fg/mL	[116]
Antibody/HRP	Tau, BACE1	6–5 × 10^3^ pg/mL, 5–10^4^ fg/mL	1.7 pg/mL, 1.67 fg/mL	[117]
Antibody/HRP	ADAM10	5.6–1.4 × 10^3^ fg/mL	0.35 fg/mL	[118]
Antibody-AP	Aβ42	10–500 pg/mL	3.9 pg/mL	[119]
Aptamer/TB-Au@COFs	AβO	0.01–180 pg/mL	4.27 fg/mL	[122]
Aptamer/MB-Au@COFs	AβO	10–10^6^ pM	5.1 pM	[121]
Antibody/SiO_2_-Au-Thi	AβO	0.05–80 ng/mL	5 pg/mL	[120]
Antibody/nanoliposomes	p-Tau181	0.1–100 pg/mL	1 pg/mL	[124]
Aptamer/AuNPs	Tau-381	0.5–10^2^ pM	0.42 pM	[125]
Antibody/AuNPs	Tau-441	0.5–80 fM	0.46 fM	[126]
Antibody/AuNPs	Tau	50–7.5 × 10^2^ ng/mL	63 ng/mL	[127]
Antibody/AuNPs	ApoE, Aβ	0–100 pg/mL	80 pg/mL, 19 pg/mL	[128]
Antibody/CuCo-CeO_2_-Pd	Aβ	1–10^5^ pM	0.05 pM	[129]
Antibody/PPy/Ni/PtNPs	Aβ42, Tau	0.1–5 ng/mL, 1–10^6^ pg/mL	0.04 ng/mL, 0.4 pg/mL	[130]
F-TiO_2_	p-Tau	10^3^–2 × 10^5^ fg/mL	1.774 pg/mL	[132]
Cd^2+^/Ti^4+^@TiP	Aβ40, p-Aβ40	1–10^5^ fM	0.45 fM	[133]
Antibody/Pb-CDs + Cu-CDs	GFAP, NFL	1–40 pg/mL	0.3 pg/mL, 0.2 pg/mL	[134]

Abbreviation: HRP, horseradish peroxidase; AP, alkaline phosphatase; TB, toluidine blue; Au@COFs, gold nanoparticle-functionalized porphyrinyl; MB, methylene blue; Thi, thionine; F-TiO_2_, flower-shaped TiO_2_; TiP, titanium phosphate nanospheres; CDs, carbon dots; BACE1, beta-secretase 1; ADAM10, α-secretase; ApoE, apolipoprotein E; GFAP, glial fibrillary acidic protein; NFL, light chain neurofilament.

## 4. ECL Biosensors

ECL is a specific chemiluminescence reaction initiated electrochemically on the electrode surface by the integration of electrochemistry and spectroscopy. ECL biosensors not only exhibit the merits of high sensitivity, wide linear range, simple operation, and easy observation of chemiluminescence, but also have many unique analytical advantages unmatched by chemiluminescence. The technique does not require an external light source and is not affected by light scattering. Since excited-state substances are generated by high-energy electron transfer reactions on the electrode, ECL biosensors show extremely high sensitivity, selectivity, and reproducibility and combine the cost-effectiveness and portability of electrochemical devices. In this method, the luminescence phenomenon can be achieved by applying a unidirectional potential scan to an electrode immersed in a solution containing luminophores and co-reactants, or by modifying the electrode with luminophores before immersing it in the co-reactant solution. Both luminophores and co-reactants can undergo oxidation or reduction reactions. The intermediates generated from oxidation or reduction reactions will further decompose into strong reducing agents or oxidants. Ultimately, chemically reactive collisions occur between the oxidized/reduced luminophores and the reducing/oxidizing agents, driving the luminophore-related compounds into excited states to emit light. ECL biosensors applied to health monitoring are usually designed through the co-reactant method. Luminophores can be mainly categorized into three types: small organic molecules, inorganic coordination complexes, and nanomaterials [135]. As for co-reactants, persulfate (S_2_ O_8_^2−^) is generally adopted for cathodic reactions, while tripropylamine (TPA) and hydrogen peroxide (H_2_ O_2_) are mainly used in anodic reactions. Luminol is a classic chemiluminescent reagent that can be applied as an ECL reagent in two forms: reaction substrate for oxidoreductase-labeled biorecognition element and labeling reagent for affinity biorecognition element. In the homogeneous luminol−H_2_ O_2_ ECL reaction system, luminol is electrochemically oxidized to generate luminol radicals, while H_2_ O_2_ undergoes electrochemical oxidation to produce HO_2_• radicals that subsequently deprotonate to form O_2_• radicals. Luminol radicals then react with HO_2_• and O_2_• radicals to form excited-state 3-aminophthalate molecules that can emit blue light with a maximum emission wavelength of 425 nm. Nevertheless, luminol molecules cannot be regenerated after electrochemical oxidation, and the ECL intensity of luminol is affected by the pH value of detection system.

Since the report of an aqueous system composed of tris(2,2′-bipyridyl)ruthenium(II) (Ru(bpy)_3_^2+^) and TPA, ECL has received widespread attention due to its ultrahigh luminescence efficiency with a peak emission wavelength of 620 nm. Homogeneous and heterogeneous ECL mechanisms for the Ru(bpy)_3_^2+^–TPA system have been extensively investigated and documented in previous literatures [136,137]. In short, the homogeneous ECL mechanism involves two reaction pathways. First, TPA and Ru(bpy)_3_^2+^ ions are directly oxidized on the electrode surface to produce TPA cation radicals. The TPA cation radicals then undergo deprotonation to produce neutral TPA radicals. Finally, neutral TPA radicals react with Ru(bpy)_3_^2+^ ions in the diffusion layer to form excited-state Ru(bpy)_3_^2+^ ions that can release photons and return to the ground state. In most ECL biosensors, Ru(bpy)_3_^2+^ derivatives are conjugated to specific sensing elements such as antibodies and DNA probes or immobilized on the microbeads. They can only stay near the electrode and cannot freely diffuse throughout the entire solution. In this case, the heterogeneous ECL mechanism is applicable, and the luminescence is triggered only by the TPA radicals diffused from electrodes. In order to label the Ru(bpy)_3_^2+^ complexes onto biorecognition elements, the bpy ligand must be functionalized with an appropriate linking group, such as carboxyl group for amine conjugation or amino group for carboxyl binding, to form a stable amide bond. In addition, as a new type of ECL materials, iridium(III) complexes have attracted increasing research interest due to their high ECL efficiency, tunable emission wavelength, and adjustable working potential through ligand structural modification. Research on iridium complexes has two primary objectives: boosting ECL efficiency and regulating their emission wavelengths and working potentials [138,139].

**Figure 8:**
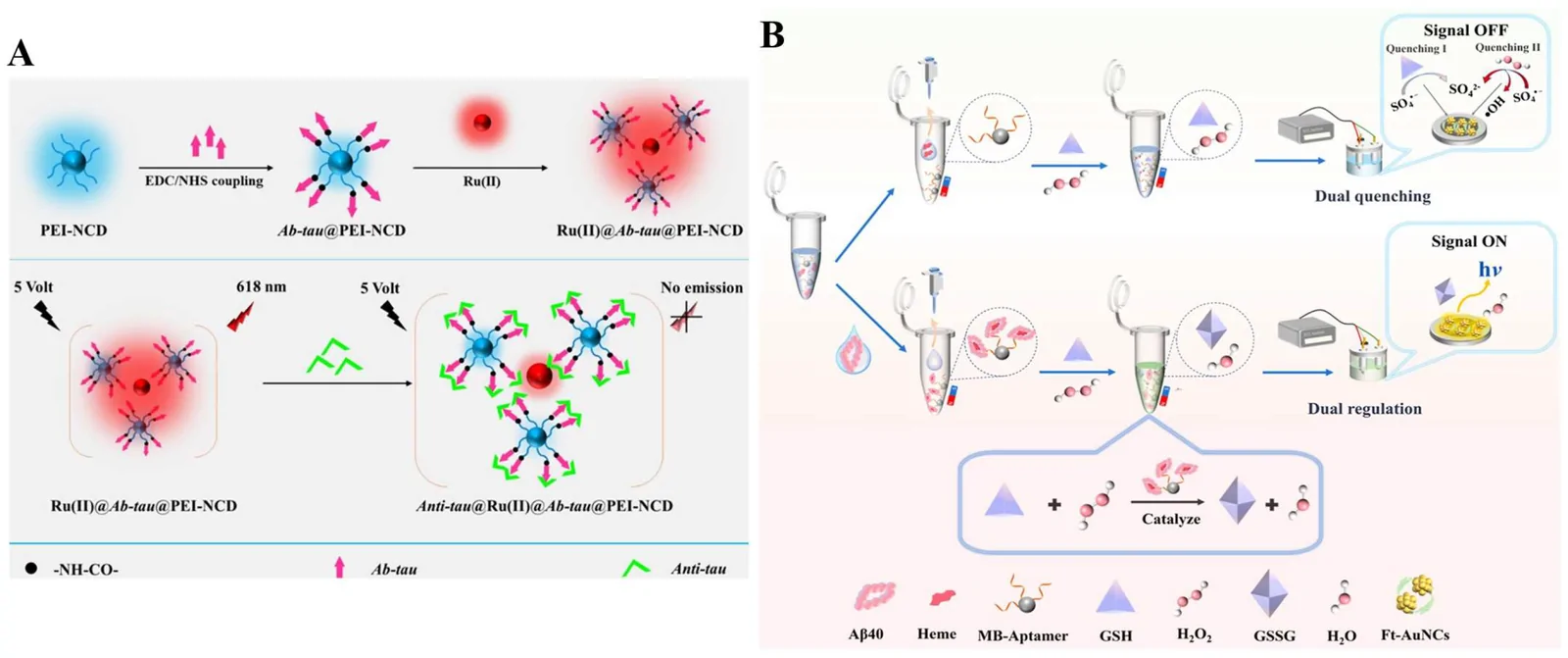
(**A**) Schematic illustration of immunoassay system for sensing of anti-Tau. Reprinted with permission from ref. [140]. (**B**) Schematic illustration of the “off-on” ECL biosensor for the detection of Aβ40. Reprinted with permission from ref. [141].

Although ECL biosensors possess have many outstanding advantages, a series of inherent defects shortcomings still need to be overcome, and their performance needs to be continuously improved to meet increasingly stringent detection requirements. For instance, conventional gold or platinum electrodes used in ECL biosensors tend to adsorb various substances from the detection solution during the testing process, resulting in unstable and transient signals. More seriously, the detection sensitivity of such biosensors is still limited. The rapid development and maturity of nanotechnologies provide abundant fabrication options for biosensors. Nanomaterials are constructed from microscale substances, and their unique surface and interface effects, quantum size effects, and macroscopic quantum tunneling effects endow them distinct adsorption, catalytic, electrochemical and surface properties compared to traditional bulk materials. Biosensors assembled from nanomaterials with diverse outstanding properties and biological recognition units integrate the physical and chemical characteristics of nanomaterials and the specific functions of biomolecules, diversifying biosensor fabrication strategies and enabling multifunctional sensing platforms. Since most superior properties of nanomaterials depends on their size and morphology, nanomaterials with customized performance can be synthesized by regulating reaction conditions during the preparation process. This adjustability enables nanomaterials to adapt in different application fields. Owing to ultrahigh luminescence intensity and excellent stability, nanomaterials are gradually replacing traditional luminophores and becoming the new generation of luminescent materials for developing ECL biosensors to diagnose AD. For example, Kala et al. developed an ECL immunosensor for Tau detection using Ru(bpy)_3_^2+^ luminophores and polyethyleneimine CDs (PEI-NCD) as the co-reactants (Figure 8A) [140]. Monoclonal anti-Tau antibodies were effectively attached onto the co-reactant PEI-NCD due to their abundant amino groups. The presence of Tau led to a decrease in the luminescence intensity. The immunosensor achieved a detection limit as low as 6.103 pg/mL with high specificity and selectivity and showed excellent recovery rates (93–104%) in spiked serum samples. He et al. proposed a sensitive ECL biosensor for plasma Aβ40 detection based on a novel “off-on” dual-regulation strategy with gold nanoclusters (AuNCs) as the luminophores (Figure 8B) [141]. The approach exploited the synergistic quenching effect of glutathione (GSH) and H_2_O_2_ on the ECL emission of AuNCs. In the absence of the target Aβ40, the quenchers remained active, thus suppressing the signal. The Aβ40-hemin complex formed between Aβ40 target and hemin showed peroxidase-like activity and catalyzed the mutual consumption of GSH and H_2_O_2_, thereby restoring the ECL signal. This target-triggered signal recovery, combined with aptamer-functionalized magnetic beads for efficient capture and separation, enabled the detection of Aβ40 with high sensitivity and specificity, offering a promising tool for non-invasive early screening of AD.

**Figure 9:**
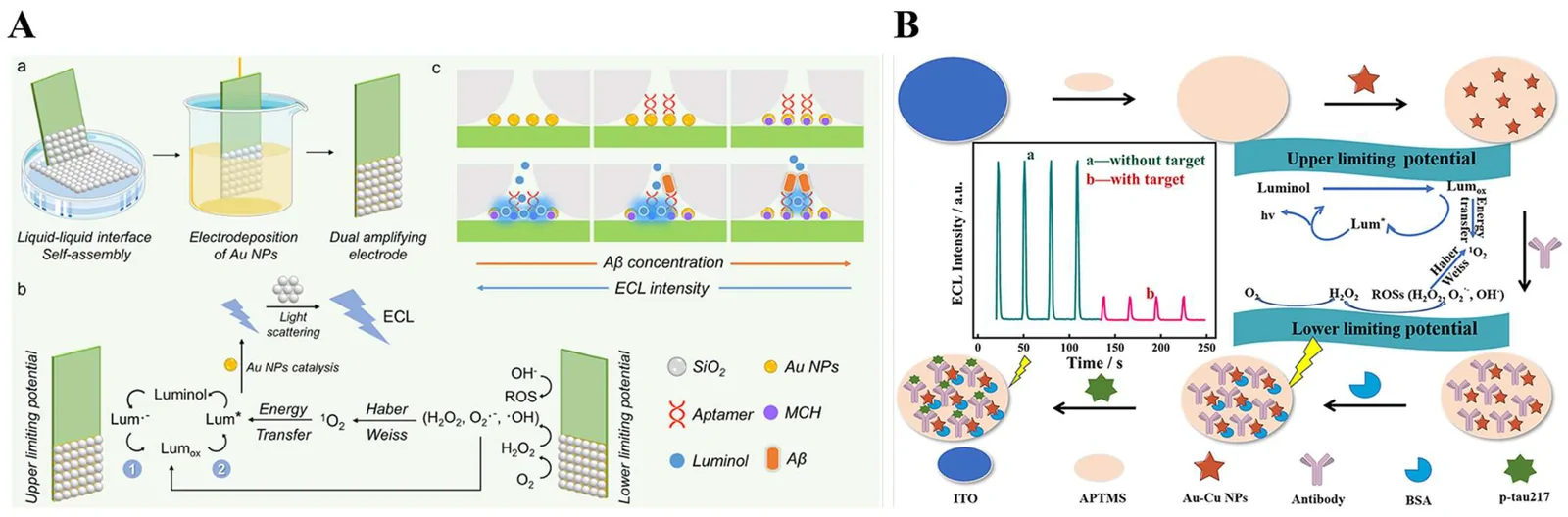
(**A**) Schematic illustration of (**a**) preparation procedure of the basal electrode with dual signal amplification for the ECL of Luminol, (**b**) ECL mechanism of luminol, and (**c**) sensing mechanism of the aptasensor for Aβ detection. Reprinted with permission from ref. [146]. (**B**) Schematic illustration of ECL immunosensor for sensing of p-Tau217. Reprinted with permission from ref. [147].

The specific combination of biorecognition elements and targets on the sensing interface may hinder the diffusion of ECL reagents to the electrode surface, resulting in reduced signal intensity. Based on this detection principle, different antibody and aptamer-based ECL biosensors have been reported for detecting Aβ and Tau proteins (Table 4) [142,143,144,145,146,147,148,149,150,151,152,153,154]. For example, Jalili et al. reported an ECL immunosensor for Tau detction using gold nanostar (AuNS)-decorated carbon nitride nanosheets (AuNS@g-CN) to improve sensing performance [142]. AuNS exhibited an enhanced ECL activity owing to its electrocatalytic and surface plasmon effect. The ECL immunosensor exhibited a linear range of 0.1~100 ng/mL with a detection limit of 0.034 ng/mL. It has been used to determine Tau in spiked serum samples with 0.972 AUC and 94.48%~108% recovery, showing promising application prospects in diagnosing AD. Tan et al. designed a label-free ECL aptasensor for determining Aβ monomer by incorporating two complementary signal enhancement strategies (Figure 9A) [146]. A close-packed monolayer of SiO_2_ particles with photonic crystal property enhanced the ECL output through light scattering, while AuNPs deposited within the interparticle gaps accelerated the oxidation of luminol emitter. This dual-amplification approach resulted in a 64-fold increase in the ECL intensity relative to a bare electrode. Upon target recognition, the captured Aβ created steric hindrance that could restrict luminol diffusion to the electrode surface, leading to a concentration-dependent decrease in the ECL signal. The aptasensor exhibited a linear response from 10 fM to 10 pM with a detection limit of 5 fM, and showed excellent selectivity against potentially interfering species, including AβO and Aβ fibrils. Validation through clinical blood samples from AD patients yielded recoveries ranging from 84% to 116%. The good consistency with ELISA results suggested the potential of this sensing platform for early-stage AD screening. In addition, Kong et al. developed a novel immunosensor for p-Tau217 detection using luminescent phenol as the ECL probe (Figure 9B) [147]. The biosensor was constructed by immobilizing Au–Cu nanoparticles on the APTMS-modified indium tin oxide (ITO) electrode by the formation of Au–N bonds. Au–Cu nanoparticles used for antibody immobilization provided a large surface area and improved the detection sensitivity. Specific binding of p-Tau217 antigen to the immobilized antibody led to a significant reduction in the ECL signal, with signal change proportional to antigen concentration. This ECL biosensor exhibited high sensitivity for p-Tau217 detection, with a linear range of 5–600 pg/mL and a detection limit of 0.56 pg/mL.

**Table 4:** Performance of direct ECL methods for the detection of AD-related blood protein biomarkers.

Electrode Material	Biomarker	Linear Range	Detection Limit	Ref.
Aptamer/THS	Aβ	0.5–5 × 10^5^ fM	0.23 fM	[143]
Aptamer/AuE	Aβ	10^–13^–10^–8^ mol/L	35 fM	[144]
Aptamer/MSM/AuNPs	Aβ	0.05–5 pM	12 fM	[145]
Aptamer/AuNPs@SiO_2_ array	Aβ	10–10^4^ fM	5fM	[146]
Antibody/Au-Cu NPs	p-Tau217	5–6 × 10^2^ pg/mL	0.56 pg/mL	[147]
Aptamer/MB-Au NCs Aptamer/MB-DNA	Aβ, Tau	0–1 nM	2.6 pM, 0.3 pM	[148]
Antibody/M_3_(HITP)_2_/AuNPs	Aβ_42_	1–5 × 10^7^ ng/mL	0.57 fg/mL	[149]
Antibody/Ru@SiO_2_ NPs	p-Tau217	1–10^5^ ng/mL	0.178 pg/mL	[150]
Aptamer/Ti_3_C_2_T_x_ MXene@MOF	Tau	0.1–10^5^ pg/mL	31.62 ag/mL	[151]
Antibody/AuNPs@CoSn(OH)_6_	Aβ40	1–8 × 10^2^ pg/mL	0.47 pg/mL	[152]
Aptamer/AuNPs@Fe-MOF	AβO	0.1–10 pM	71 fM	[153]

Abbreviation: THS, triple-helix DNA system; AuE, gold electrode; MSM, mesoporous silica membrane; AuNPs, gold nanoparticles; NPs, nanoparticles; NCs, nanoclusters; MB, methylene blue; M_3_(HITP)_2_, M_3_(Hexaiminotriphenylene)_2_ (M = Co, Fe, Ni); MOF, metal-organic framework.

**Figure 10:**
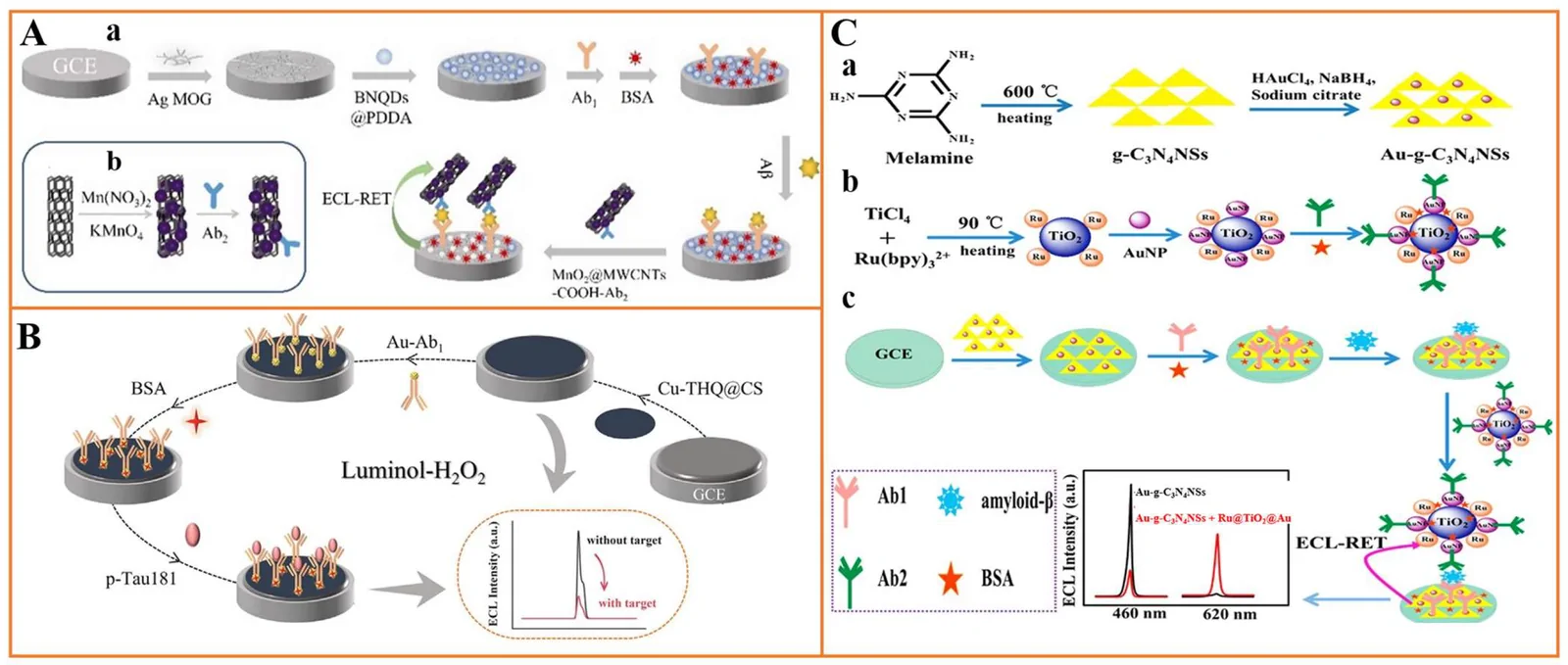
(**A**) Schematic illustration of (**a**) construction process of the ECL immunosensor, and (**b**) preparation of the quenching probe. Reprinted with permission from ref. [155]. (**B**) Schematic illustration of the construction process of the ECL immunosensor for p-Tau181 detection [156]. (**C**) Schematic illustration of (**a**) preparation of Au-g-C_3_N_4_NSs, (**b**) Fabrication of Ru@TiO_2_@Au-Ab_2_ conjugate, and (**c**) assembly process of DWR-ECL immunosensor. Reprinted with permission from ref. [157].

One of the greatest challenges in screening AD is the interference from non-target substances in biological fluids. Biomarkers such as Aβ and Tau are present at extremely low concentrations in blood and often coexist with structurally similar proteins or degradation products, increasing the diagnosis risk of cross-reactions. Although the direct detection mode for ECL biosensors is simple, its sensitivity is far from meeting current clinical needs. Therefore, researchers have focused on introducing various signal amplification strategies and detection modes into the development of ECL biosensors. For example, different nanomaterials, such as mesoporous silica, metal–organic frameworks, covalent organic frameworks, and liposomes, have been used to load ECL luminophores, nanocatalysts, and other signaling elements to enhance the analytical performance of ECL biosensors due to their large specific surface area, easy modification, and good biocompatibility (Table 5) [155,156,157,158,159,160,161,162,163,164,165,166]. Surface passivation techniques such as treatment with bovine serum albumin, polyethylene glycol, or zwitterionic layers have been successfully employed to reduce non-specific adsorption and enhance anti-interference capability. Typically, Sheng et al. reported an ECL immunosensor for ultrasensitive detection of Aβ42 based on a ternary system with boron nitride quantum dot (BNQD) as the emitter, K_2_S_2_O_8_ as the co-reactant, and AgMOG as the co-reaction accelerator (Figure 10A) [155]. AgMOG was employed to enhance the ECL signal by catalyzing the reduction of S_2_O_8_^2–^ to generate more SO_4_^•–^ radicals and load abundant BNQD and capture antibody via the Ag-N interaction. PDDA was added as a stabilizer, achieving a 31-fold signal enhancement. MnO_2_@MWCNT served as an energy acceptor, enabling efficient ECL–RET due to the spectral overlap with BNQD emission. In this sandwich immunoassay, Ab_1_-modified electrode was used for Aβ42 capture, and Ab_2_-MnO_2_@MWCNT was employed as the quenching probe. The ECL signal decreased proportionally to the target concentration with a linear range from 10 fg/mL to 100 ng/mL and a detection limit of 2.89 fg/mL, along with excellent selectivity, stability, and reproducibility. For the assays of Aβ42 in human serum samples, the recovery rates ranged from 94.2% to 103.5%, indicating practical applicability of this method. Meanwhile, Sheng et al. reported a super-enhanced cathodic ECL system for luminol in a neutral solution using copper tetrahydroxyquinone (Cu-THQ) MOF as the co-reaction accelerator (Figure 10B) [156]. The enhancement relied on a dual-catalytic mechanism of Cu-THQ. The Cu-O_4_ active sites catalyzed the decomposition of H_2_O_2_ to generate abundant reactive oxygen species, including hydroxyl radicals (•OH) and superoxide radicals (•O_2_^–^), which could oxidize luminol into the excited species. Then, Cu-THQ promoted the electrolysis of water at the electrode surface, generating a localized alkaline microenvironment with pH rising from 7.4 to 12.1. The in situ elevated pH facilitated the deprotonation of luminol intermediates, a critical step for efficient ECL emission that normally requires bulk alkaline conditions. Through these two synergistic effects, the system achieved a 4500-fold enhancement in cathodic ECL signal with excellent stability, with an emission onset at –1.30 V and a peak wavelength at 437 nm. Based on this ECL system, a label-free immunosensor was established for p-Tau181 detection with a linear range from 50 fg/mL to 100 ng/mL and a detection limit of 4.56 fg/mL, along with good selectivity, stability, and reproducibility. In human serum analysis, recovery rates ranged from 93.03% to 104.87%, confirming the reliability of this method for complex biological samples. Dual-signal ratiometric detection, multi-step washing processes, and nanostructured surfaces maximize selective binding between biosensor components, effectively reducing contamination and improving signal-to-noise ratio and selectivity. Qin et al. developed a dual-wavelength ratiometric ECL biosensor for Aβ42 detection using Au-g-C_3_N_4_ nanosheets as the donors and Ru@TiO_2_@Au composites as the acceptors (Figure 10C) [157], proposing an efficient dual-wavelength ratiometric ECL method. This method showed a linear range of 10 fg/mL–200 ng/mL and a detection limit of 2.6 fg/mL.

**Figure 11:**
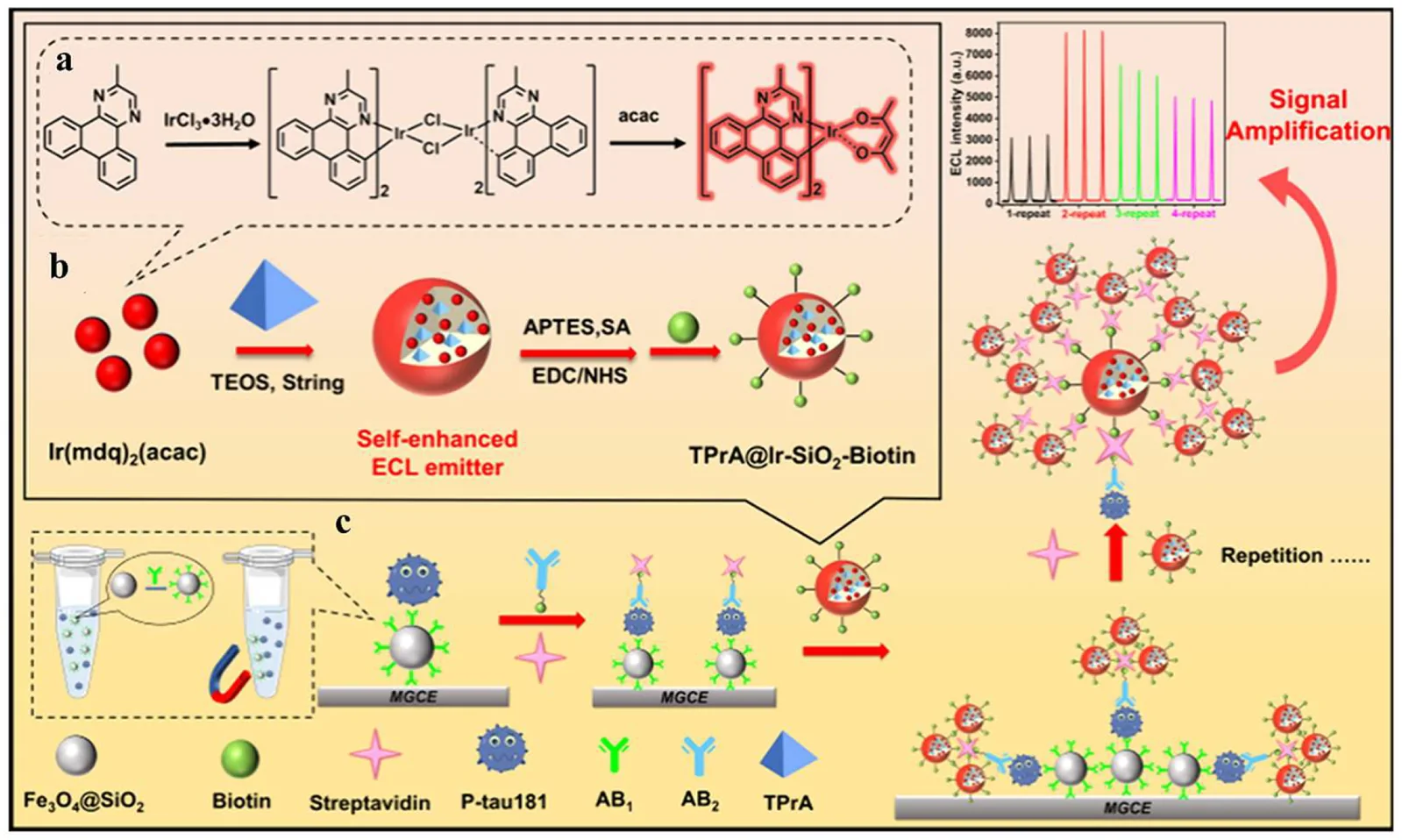
Schematic illustration of (**a**) synthetic route for Ir(mdq)_2_, (**b**) preparation of TPrA@Ir-SiO, and (**c**) ultrasensitive ECL magnetic immunosensor to efficiently detect biomarkers of AD. Reprinted with permission from ref. [159].

The localized surface plasmon resonance effect of plasmonic materials can significantly enhance the signal intensity and sensitivity of ECL detection. The technique, known as surface-enhanced ECL, has been widely applied in ECL biosensors. However, the effect of surface-enhanced ECL is restricted by wavelength, distance between luminophores and plasmonic materials, and the structure of plasmonic materials [167,168]. In addition, the surface plasmon resonance effect produced by noble metal monomers (such as AuNPs and gold dendrimers) is not ideal and cannot effectively enhance ECL signals. Therefore, it is of great significance to research plasmonic materials with high surface plasmon resonance effect. Silica nanoparticles, with excellent structural properties as well as high chemical and thermal stability, are often used to encapsulate ECL luminophores and co-reactants, showing promising prospects in the field of ECL detection of disease biomarkers. Dai et al. designed an iridium-based self-enhanced ECL emitter (TPA@Ir-SiO_2_) by incorporating Ir(mdq)_2_(acac) and its co-reactant TPA into SiO_2_ nanoparticles, significantly amplifying the initial ECL signal (Figure 11) [159]. On this basis, an ultrasensitive ECL magnetic immunosensor was developed for p-Tau181 detection based on a sandwich immunoassay format. Specific and high-affinity interaction between streptavidin and biotin further promoted the signal amplification effect. The multiple amplification strategy combining self-enhanced ECL with specific and high-affinity interactions effectively improved the sensitivity and selectivity, with a linear range of 0.1 pg/mL–0.1 μg/mL. The reliability of the sensor was verified by spiked serum sample tests, with recovery rates of 98–104%. This work demonstrated the considerable potential of the multi-signal amplification strategies in creating efficient ECL immunosensors for AD diagnosis.

**Figure 12:**
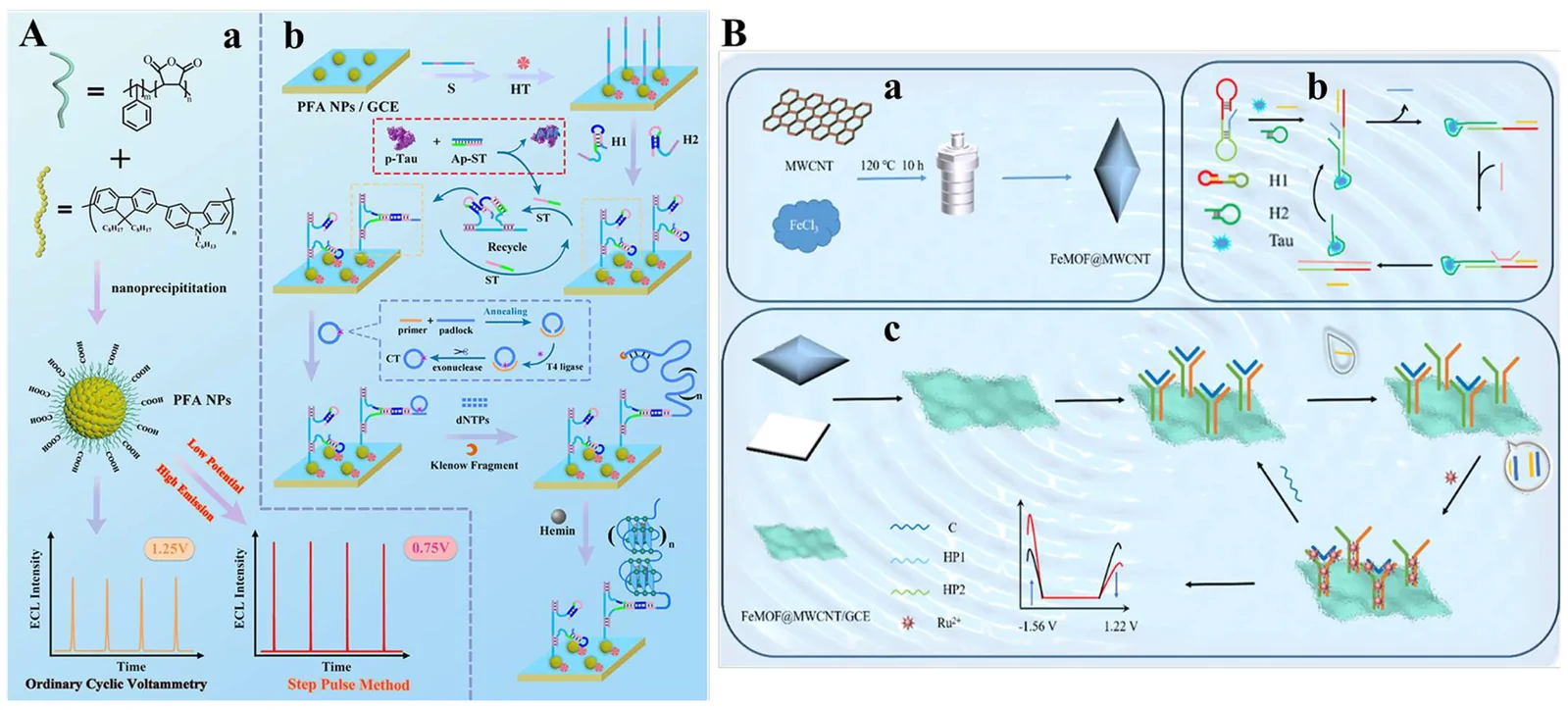
(**A**) Schematic illustration of (**a**) preparation of PFA NPs and (**b**) construction of ECL biosensor. [170] (**B**) Schematic illustration of (**a**) synthesis of FeMOF@MWCNT, (**b**) process of CHA, and (**c**) ratiometric ECL biosensor for Tau detection. Reprinted with permission from ref. [171].

The design of biological interfaces for ECL biosensors used to detect specific targets is an extremely critical research topic, as it will directly decide the accuracy, sensitivity, and applicable range of biosensors. The core challenge lies in cleverly integrating target analytes, biorecognition elements, ECL reagents or nanoprobes, as well as electrodes or magnetic beads to form complete sensing systems. Traditional methods commonly used to construct biological interfaces for electrochemical and optical biosensors are also applicable to fabricate ECL sensing interfaces and signal labels. In addition, the aforementioned nucleic acid-based signal amplification strategies for the construction of electrochemical biosensors can also be used to design ECL biosensors with high sensitivity [162,169,170,171]. For example, Zhao et al. reported an ECL biosensor for p-Tau protein with a step pulse (SP) technique to lower the triggering potential of polyfluorene (PFA) nanoparticles from 1.25 V to 0.75 V (Figure 12A) [170]. The SP mode could accelerate radical generation and reaction through rapid potential switching, reducing energy loss and producing more photons. Aptamer recognition was integrated with CHA and RCA technique to generate long DNA chains that could form G-quadruplex/hemin complexes to consume H_2_O_2_ and restore the quenched ECL signal. The biosensor exhibited a linear range from 5 fg/mL to 1 ng/mL with a detection limit of 4.15 fg/mL. In clinical serum analysis, recovery rates ranged from 97.3% to 105%, showing good agreement with ELISA and exhibiting great potential for AD diagnosis. In addition, Yuan et al. reported a ratiometric ECL biosensor for Tau protein using machine learning to guide the synthesis of an iron-based MOF (FeMOF) as a bidirectional regulator for Ru(bpy)_3_^2+^ luminophore (Figure 12B) [171]. The FeMOF enhanced the cathode ECL signal and suppressed the anode signal through the electron transfer pathway, making the ratiometric output more stable and accurate. Target recognition triggered the CHA reaction, and Ru(bpy)_3_^2+^ was loaded onto the DNA strand through rapid electrodeposition. The biosensor showed a linear range of 10–10^6^ fg/mL with a detection limit of 3.38 fg/mL, and exhibited excellent selectivity, stability over 21 days, and good reproducibility. Recoveries for the assays of clinical serum samples ranged from 96.5–107.6%. The blind testing of 20 samples showed 80% specificity and 90% sensitivity, demonstrating great potential for clinical diagnosis of AD.

**Figure 13:**
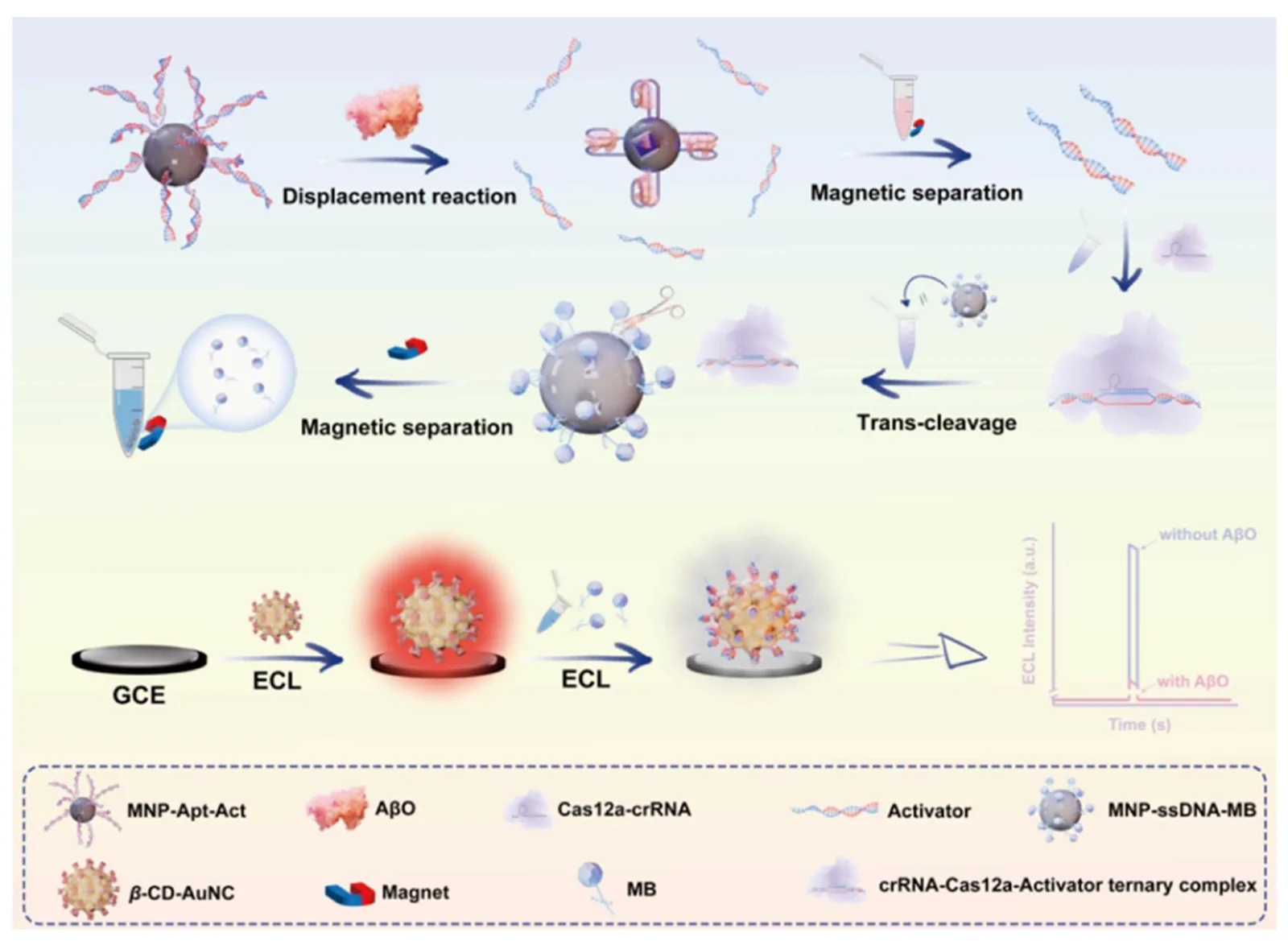
Schematic illustration of a split-type ECL analysis platform based on the CRISPR/Cas12a system for AβO detection. Reprinted with permission from ref. [173].

Magnetic materials can act as carriers to immobilize biomarkers, facilitating the separation and detection of low-abundance biomarkers. These characteristics have driven the emergence of various sensitive, high-throughput automated immunoassay methods. Yan et al. reported a synergistic terminal deoxynucleotidyl transferase (TdT)/Clostridium butyricum Argonaute (CbAgo)-mediated protein-to-nucleic acid conversion strategy, effectively converting the antibody recognition event of Aβ42 into a nucleic acid-assisted signal-amplified output [172]. In this system, Aβ42 was specifically enriched by magnetic immunocapture units (MNPs-Ab_1_) and then conjugated with signal transduction units (Ab_2_-DNA) to form protein–nucleic acid magnetic nanocomposites. TdT and CbAgo initiated the cyclic “extension-cleavage” reactions on the single-stranded DNA, generating abundant free DNA fragments as amplifiable intermediates. Quantitative analysis of the released DNA using a switchable ECL biosensor integrating with CRISPR/Cas12a system achieved a detection limit of 2.79 fg/mL for Aβ42. This biosensor has been successfully applied to the assays of artificial biological fluids, SH-SY5Y cell-derived exosomes, and clinical serum samples, providing a powerful and versatile biosensing method for trace biomarker analysis and early clinical diagnosis of AD. In addition, Li et al. developed an amplification-free split-type ECL biosensing platform by integrating CRISPR/Cas12a, host–guest chemistry, and β-cyclodextrin (β-CD)-modified AuNCs for ultrasensitive detection of AβO (Figure 13) [173]. Target-triggered Cas12a cleavage induced the release of MB tags from MNPs, quenching the ECL signal of β-CD-AuNCs via electron transfer upon the host–guest recognition. The platform achieved a detection limit of 0.2 fg/mL (3–4 orders lower than ELISA) with excellent selectivity and reproducibility. Clinical validation using AD serum samples showed good recovery (99.6–106.0%) and comparable correlation with ELISA, demonstrating the reliability of this method for early diagnosis of AD.

**Table 5:** Performance of sandwich-type ECL methods for the detection of AD-related blood protein biomarkers.

Signal Tag	Biomarker	Linear Range	Detection Limit	Ref.
Ru@TiO_2_@AuNPs	Aβ42	10^−5^–2 × 10^2^ ng/mL	2.6 fg/mL	[157]
Ru(bpy)_3_^2+^@SiO_2_ NPs	AβO	50–10^7^ fM	14 fM	[158]
TPrA@Ir-SiO_2_	P-Tau181	0.1–10^5^ pg/mL	68.58 fg/mL	[159]
PFA NPs/DNA/AgNPs	p-Tau 181	10–5 × 10^7^ fg/mL	6.9 fg/mL	[160]
PFA NPs/DNA/AuNPs	AβO	10–10^6^ nM	8.3 fM	[161]
Cu NCs@3D-LDH/AgNPs/Fc-DNA	GFAP	10–10^8^ pg/mL	2 ag/mL	[162]
MXene-Pd@Pt/AuNPs	AβO	0–10 nM	25 fM	[163]
CdS QDs/AuNPs	AβO	10–10^7^ nM	3.1 fM	[164]
MoS_2_@Co_SA_/UiO-66	p-Tau	20–10^6^ ng/mL	7.2 fg/mL	[165]
PSP NPs	Tau	10–10^3^ fg/mL	5.45 ag/mL	[169]
PFA NPs	p-Tau	5–10^6^ ng/mL	4.15 fg/mL	[170]
Ru(bpy)_3_^2+^/FeMOF@MWCNT	Tau	10–10^6^ fg/mL	3.38 fg/mL	[171]
β-CD-AuNCs/MB	AβO	10–10^8^ ng/mL	0.2 fg/mL	[173]

Abbreviation: AuNPs, gold nanoparticles; NPs, nanoparticles; FeMOF, iron-based metal-organic framework; MWCNT, multi-walled carbon nanotube; TPrA, PFA, poly[(9,9-dioctyl-fluorenyl-2,7-diacyl)-alt-co-(9-hexyl-3,6-carbazole)]; AgNPs, silver nanoparticles; TPrA, tri-n-propylamine; Ir, iridium-based luminophores; 3D-LDH, 3D layered double hydroxide; Fc, ferrocene; Co_SA_, Co single-atom; UiO-66, zirconium-based metal-organic framework; PSP, 2,5-diphenyl-1,2,4-oxadiazole functionalized by sodium dodecyl sulfate and polyethylenimine; β-CD, β-cyclodextrin; AuNCs, gold nanoclusters; MB, methylene blue. GFAP, Glial fibrillary acidic protein.

## 5. PEC Biosensors

PEC bioanalysis is an emerging detection technique with excellent performance and ease of use. Its detection principle relies on the change in photocurrent upon the interaction between recognition element and target analyte. Since the excitation light and electrical signal correspond to different energy forms, PEC biosensors exhibit higher sensitivity and signal-to-noise ratio than traditional electrochemical methods. In addition, their simple electronic readout system contributes to the miniaturization of sensing devices. Owing to these advantages and great potential of PEC bioanalysis, extensive research has focused on the detection of AD-related protein biomarkers with this technique [174]. The biorecognition event can alter the photocurrent by affecting the electron transfer, charge separation, or steric hindrance at the electrode interface, enabling highly sensitive detection.

Improving the sensitivity of PEC biosensors in complex biological samples requires the development of solutions. High photoelectric conversion efficiency materials for signal transmission or amplification are required. Existing photoelectric materials or films have many limitations in practical applications, such as low photoelectric conversion efficiency, poor stability, and unsatisfactory reproducibility. For example, titanium dioxide (TiO_2_) is widely used in PEC sensors due to its controllable morphology, strong photocatalytic activity, good chemical stability, and biocompatibility; however, it can only be activated by ultraviolet light (bandgap of 3.2 eV), limiting its sunlight utilization efficiency. In addition, ultraviolet irradiation may cause irreversible damage to biomolecules. Therefore, developing non-toxic, low-cost photosensitive materials that can efficiently utilize visible light is crucial for the development of PEC biosensors. Recently, various nanomaterials have been developed and used as photoactive electrode materials [175,176,177,178]. For example, graphitic carbon nitride (g-C_3_N_4_), a low-cost, metal-free semiconductor material with a bandgap of 2.7 eV, shows excellent photochemical stability, suitable bandgap for visible light absorption, and good environmental performance. MXene, a family of two-dimensional transition metal carbides, nitrides, or carbonitrides, is a predominantly conductive material to enhance the photocurrent generated by PEC sensors.

The quantitative detection of biomarkers is generally based on the photocurrent variations induced by specific interactions between the target analytes and the photosensitive substrates/probes. The concentrations of AD biomarkers are extremely low in the early pathological stages. The intrinsic properties of photoelectrode materials directly decide the magnitude of the photocurrent generated by the PEC systems, thereby exerting a significant impact on the sensitivity. The selection of photoelectrode materials requires a balance between the photoelectric conversion efficiency and the biomolecule immobilization ability. Up to now, various photoelectrode materials including semiconductors, metals, and carbonaceous materials have been successfully applied to fabricate PEC biosensors [179]. Semiconductor nanomaterials that can convert light into electrical signals play a pivotal role in constructing PEC biosensors. Multiple semiconductor materials with distinct energy band structures and electron transport mechanisms are available for the design of PEC biosensors. Nevertheless, single-component materials often fail to satisfy the practical analytical demands due to their inherent performance limitations. Therefore, composites with unique electronic band structures have become important photoelectric conversion materials with enhanced photocatalytic activity. Such composites can improve the photon-to-current conversion efficiency, boost the electrical conductivity, and strengthen the immobilization of biomolecules. Multiple signal amplification strategies based on photoelectric materials have been developed to achieve ultrasensitive detection of biomolecules, including inorganic-inorganic composites, inorganic-organic heterostructures, semiconductor-metal hybrids, semiconductor-carbon composites (carbon nanotubes, mesoporous carbon, graphene), and semiconductor-upconversion composites [180]. For example, Gao et al. constructed a PEC biosensor for AβO detection by integrating self-enhancing photoactive substrates of CdS/ZnS/Bi_2_Se_3_ ternary hybrids with PtCu nanoflowers as dual quenchers [181]. DNA cascade-activated walker was used for signal amplification with a detection limit of 4.4 fM. Reliable quantification of AβO in blood samples from AD patients was achieved with excellent specificity and stability. Wang et al. developed a light-addressable PEC immunosensor array for high-throughput detection of Tau by employing in situ grown Ag_2_S@ZnO nanorod as the photoactive material and Au@CeO_2_ as the signal suppressor [182]. This work incorporated a self-calibration strategy to minimize inter-site variability, attaining a detection limit of 1.5 pg/mL and showing promising applicability in clinical serum analysis.

Steric hindrance effect has become an important method widely applied in PEC biosensors. Biocatalytic precipitation and bioconjugation are the two major approaches for steric hindrance-based PEC biosensors [183,184,185]. Both of the methods can amplify photocurrent by obstructing the transport and diffusion of electrons, donors, and acceptors to the photoelectrode surface. Two detection modes can be adopted in this process: signal-on and signal-off. The commonly used detection formats in PEC biosensors are based on the formation of direct or sandwich-type antigen-antibody immunocomplexes. Insoluble precipitates formed on electrode surfaces can hinder the transfer of mass and electrons, leading to a sharp drop in the photocurrent signal. Zhang et al. developed a label-free PEC immunosensor for Aβ detection using SnO_2_/CdCO_3_/CdS nanocomposites [186]. The hierarchical band arrangement of the nanocomposites facilitated efficient electron transfer from CdS through CdCO_3_ to SnO_2_, drastically suppressing charge recombination and yielding a photocurrent far superior to that of its individual components. This one-pot hydrothermal method for synthesis of the nanocomposites avoided the tedious multi-step procedures in the preparation of composite materials. The immunosensor was designed based on a signal-off principle, where the immunocomplex formed between anti-Aβ and Aβ blocked the electron transfer, enabling a wide linear range and a low detection limit. This work provided a straightforward and effective strategy for constructing high-performance PEC biosensing platforms. In addition, Li et al. constructed an Au-C_3_N_4_ decorated TiO_2_/ZnS type-II heterojunction PEC aptasensor to resolve the low sensitivity and severe non-specific interference of traditional PEC biosensors for the detection of trace plasma Aβ42 [187]. This design adopted TiO_2_/ZnS staggered heterojunction to optimize band alignment and separate photogenerated carriers. Au-loaded g-C_3_N_4_ serving as an electron transfer mediator was further integrated to broaden visible light absorption via local surface plasmon resonance effect, greatly preventing carrier recombination and amplifying photocurrent signal. Thiol-modified Aβ42-specific aptamer was immobilized on the gold site through the formation of Au-S bond, and 6-mercapto-1-hexanol (MCH) was applied to block redundant electrode sites and eliminate protein fouling in complex plasma matrices. The specific binding of Aβ42 with the aptamer led to an increase in the steric hindrance, suppressing interfacial charge transfer and producing a measurable reduction of photocurrent for quantitative detection. This platform achieved a detection limit of 127.4 fg/mL with a wide linear range from 10^–16^ to 10^–12^ g/mL, and exhibited excellent anti-interference capability against Aβ40, Tau, glucose, and other biomolecules with outstanding long-term stability. For the assays of clinical plasma samples from AD patients, the results were highly consistent with the gold-standard single-molecule array method, showing small relative standard deviation and reliable clinical accuracy. This antifouling, ultrasensitive PEC aptasensor provided a portable, low-cost strategy for minimally invasive early screening of AD.

**Figure 14:**
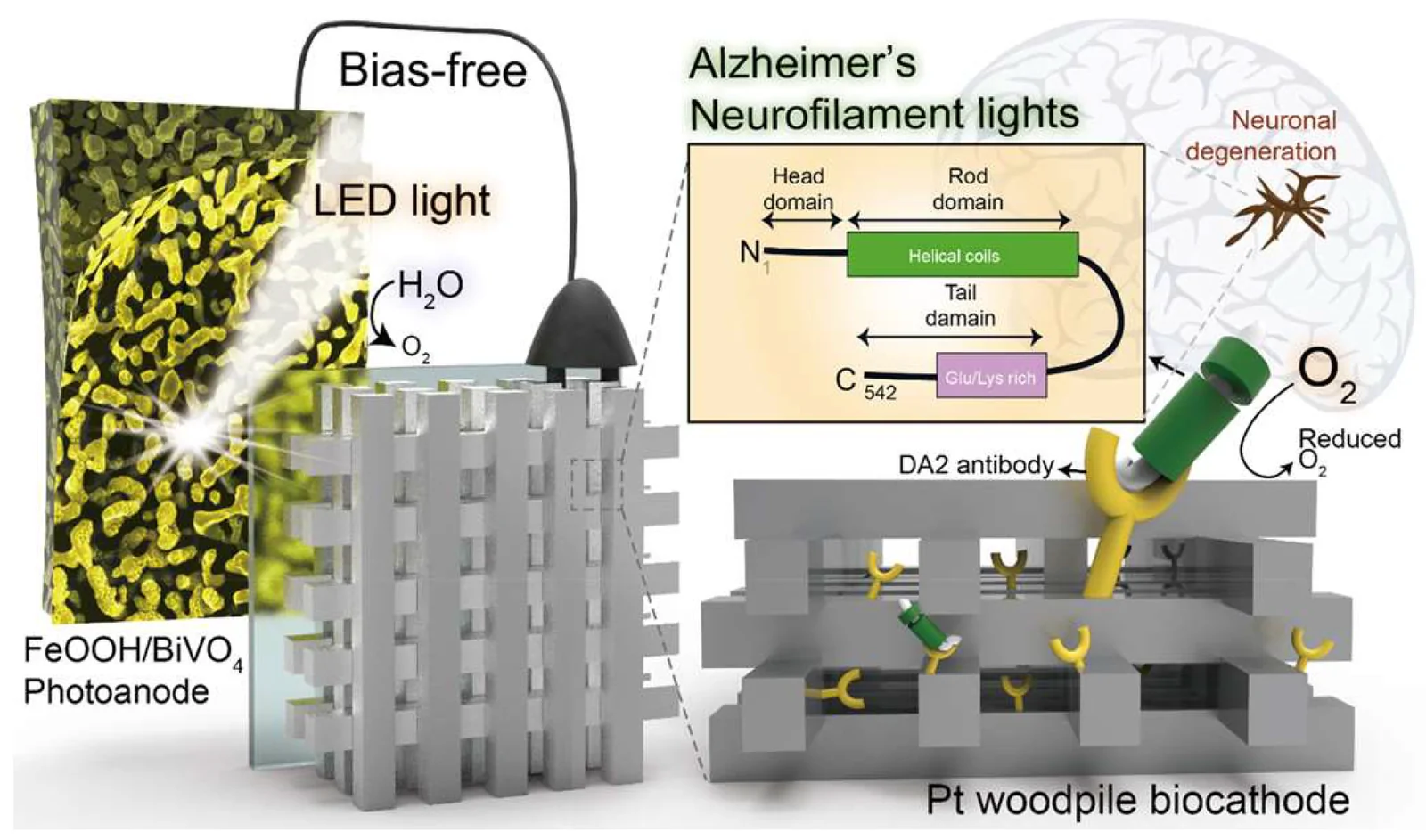
Schematic illustration of unbiased PEC sensing of AD NFL. Reprinted with permission from ref. [189].

Self-powered sensor platforms can eliminate the need for external power sources, facilitating device miniaturization and cost reduction. For this consideration, Hu et al. addressed the critical challenge of biofouling in PEC immunoassays and developed a immunosensor for Aβ40 detection based on a Z-scheme PEDOT/ZnIn_2_S_4_ heterojunction and a zwitterionic peptide [188]. The heterojunction formed between the conductive polymer PEDOT and ZnIn_2_S_4_ promoted efficient charge separation and generated a strong photocurrent. In order to suppress non-specific protein adsorption in complex biological media, the authors employed a zwitterionic peptide as a blocking agent to form a hydrophilic and electrically neutral hydration layer at the sensor interface. The signal-off mechanism based on the increased steric hindrance from specific immunocomplex formation ensured high selectivity and low detection limit, demonstrating the practical utility of this antifouling strategy for protein detection in clinical samples. In addition, Kim et al. constructed a self-powered, bias-free PEC sensing platform using a stake-shaped platinum nanowire array cathode and an oxyhydroxide-deposited bismuth vanadate (FeOOH/BiVO_4_) photoanode for NFL detection (Figure 14) [189]. Platinum-based stake-shaped nanostructures were adopted due to their abundant reduction-active sites and easy of oxygen mass transfer. Coupling stake-shaped platinum nanowire array conjugated with NFL-specific antibody with water-oxidizing FeOOH/BiVO_4_ photoanode enabled sensitive detection of plasma NFL with a detection limit of 38.2 fg/mL.

Redox cycling system can generate reusable signal reporters, which has been verified as an efficient signal amplification method for electrochemical and PEC bioanalysis [190]. Wang et al. reported a self-powered PEC immunosensor for Aβ detection by using NH_2_-UiO66/TAPA-DHNDA-COF heterojunction as the photocathode and NiCo_2_S_4_@ZnIn_2_S_4_ as the photoanode [191]. CdS-QDs and CdTe-QDs were employed as the signal tags for Aβ40 and Aβ42, respectively. The method had detection limits of 0.33 fg/mL for Aβ40 and 0.24 fg/mL for Aβ42 with a linear range of 1~10^6^ fg/mL. Recovery experiments were performed by spiking the targets in artificial CSF and plasma from healthy controls and MCI patients, providing a promising tool for early diagnosis AD.

Energy transfer between semiconductor donors (CdS, CdSe, CdTe) and metallic nanoparticle acceptors (Au/Ag NPs) has been proven to be an effective strategy for expanding the range of signal variation. The transfer relies on the spectral overlap between the broad absorption bands of metal nanoparticles and the emission spectra of quantum dots (QDs). At short interparticle distances, the luminescence of QDs can excite the localized surface plasmon resonance in metal nanoparticles and attenuate the photocurrent derived from QDs. Direct conjugation of metal nanoparticles to bioprobes will only cause weak photocurrent changes due to the limited binding sites on biomolecules. The combination of metal nanoparticles with high-surface-area functional materials can improve the loading capacity of nanoparticles and amplify the magnitude of photocurrent variations [182,192,193]. Wang et al. reported two complementary platforms for Aβ42 detection: light-addressable PEC immunosensor array and PEC-lateral flow immunoassay strip [182]. CdTe-QD-sensitized UiO-66 MOF and PCN-224@ZnIn_2_S_4_ heterojunction were used as the sensing matrix and immunoprobe, respectively. The array achieved a low detection limit (3.1 fg/mL) and the strip enabled rapid, point-of-care quantification within 15 min, validated in artificial CSF and human plasma. Zhang et al. presented a cathodic PEC aptasensor for AβO detection by employing black phosphorus quantum dot (BPQD) as the photocathode and gold nanorod for surface plasmon resonance-enhanced signal amplification through a dual-aptamer sandwich format. The method has a broad linear range from 10 fM to 100 nM with a detection limit of 4.21 fM for analyzing human serum samples [193].

**Figure 15:**
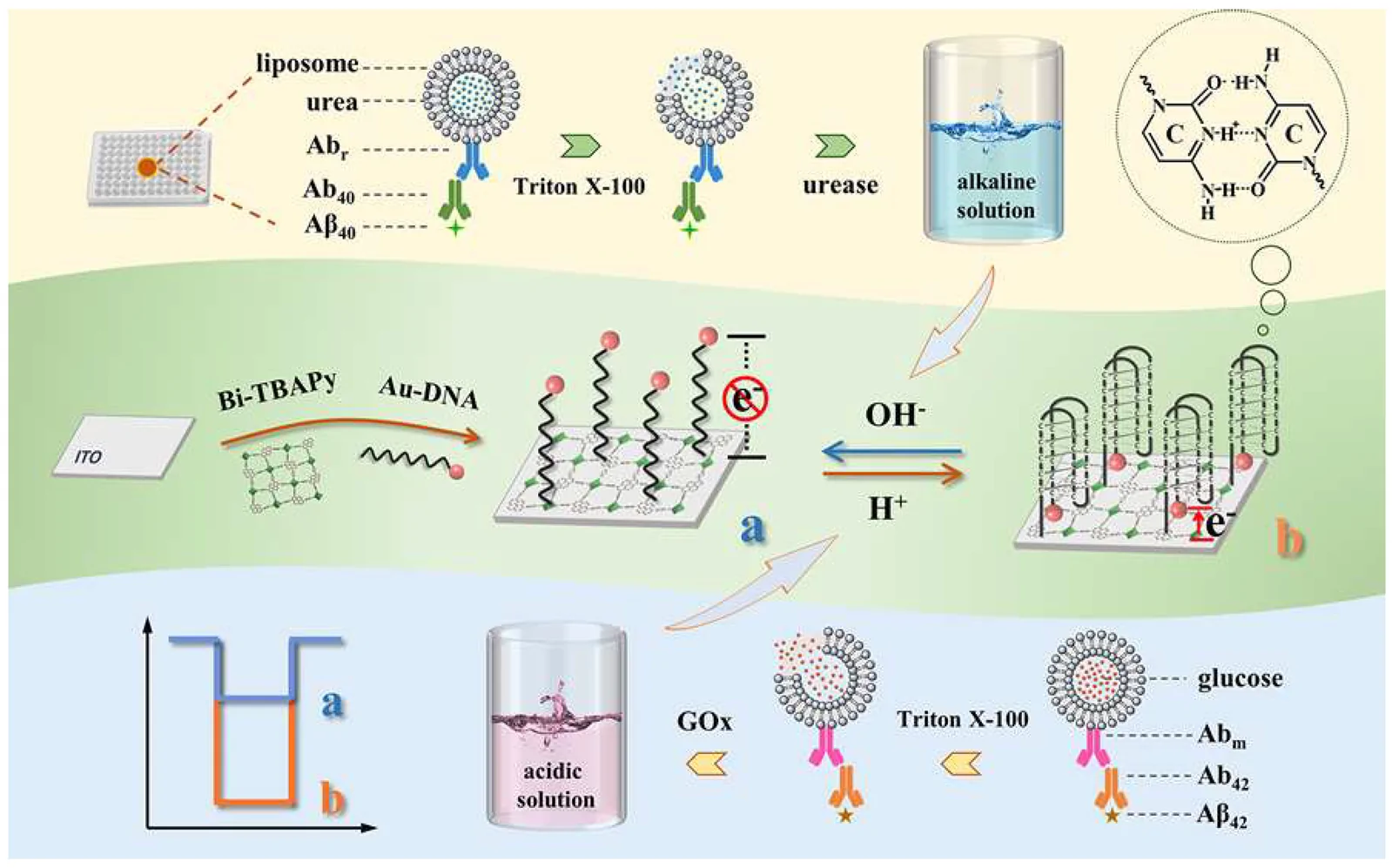
Schematic illustration of the pH-regulated switchable multiplex PEC sensing mechanism for Aβ42 and Aβ40. Reprinted with permission from ref. [194].

Electron donors/acceptors serving as efficient hole and electron scavengers can amplify photocurrent responses and improve overall PEC performance. The detection sensitivity of PEC biosensors can be improved by rationally adjusting the dosage of electron donors and acceptors. Generally, the designed recognition systems are built on the basis of large-scale production of electron donors/acceptors through labeling or loading approaches, involving two mainstream pathways: generating electron donors/acceptors with the aid of enzymes and releasing electron donors/acceptors from nanocarriers. Enzyme-assisted strategies refer to the use of natural enzymes or synthetic nanozymes to catalyze corresponding substrate reactions so as to produce substances that can act as electron donors or acceptors. These substances can trap photogenerated electrons and holes and effectively suppress electron-hole recombination, thus significantly amplifying photocurrent signals. The conjugation of enzymes or nanozymes with antibodies enables in-situ generation of electron donors/acceptors to further enhance photocurrent responses. In addition, lysis with Triton X-100, enzymatic hydrolysis, nucleic acid hybridization or acid etching can trigger the release of electron donor/acceptor substances encapsulated in liposomes, ferritin, nucleic acid hybrids, and silica nanospheres, effectively realizing signal amplification. The released electron donors/acceptors can facilitate hole-trapping reactions and strengthen photocurrent signals. For example, Gao et al. reported a multiplex PEC immunoassay platform for simultaneous detection of Aβ40 and Aβ42 using pH-responsive i-motif DNA probes (Figure 15) [194]. The pH-modulated conformational switch of the i-motif structure regulated the electron-transfer tunneling distance between AuNPs tags and Bi-TBAPy MOF-modified photocathode. Target-dependent enzymatic reactions in a split-type 96-well plate led to acidic or alkaline solutions, triggering the formation or unraveling of the i-motif and consequently switching the photocurrent “ON” or “OFF”. This strategy overcame the traditional dependence on wavelength or multiplexing potential, providing a simplified and accurate platform for determining the Aβ_42_/Aβ_40_ ratio, a more reliable indicator for distinguishing AD from other types of dementia.

**Figure 16:**
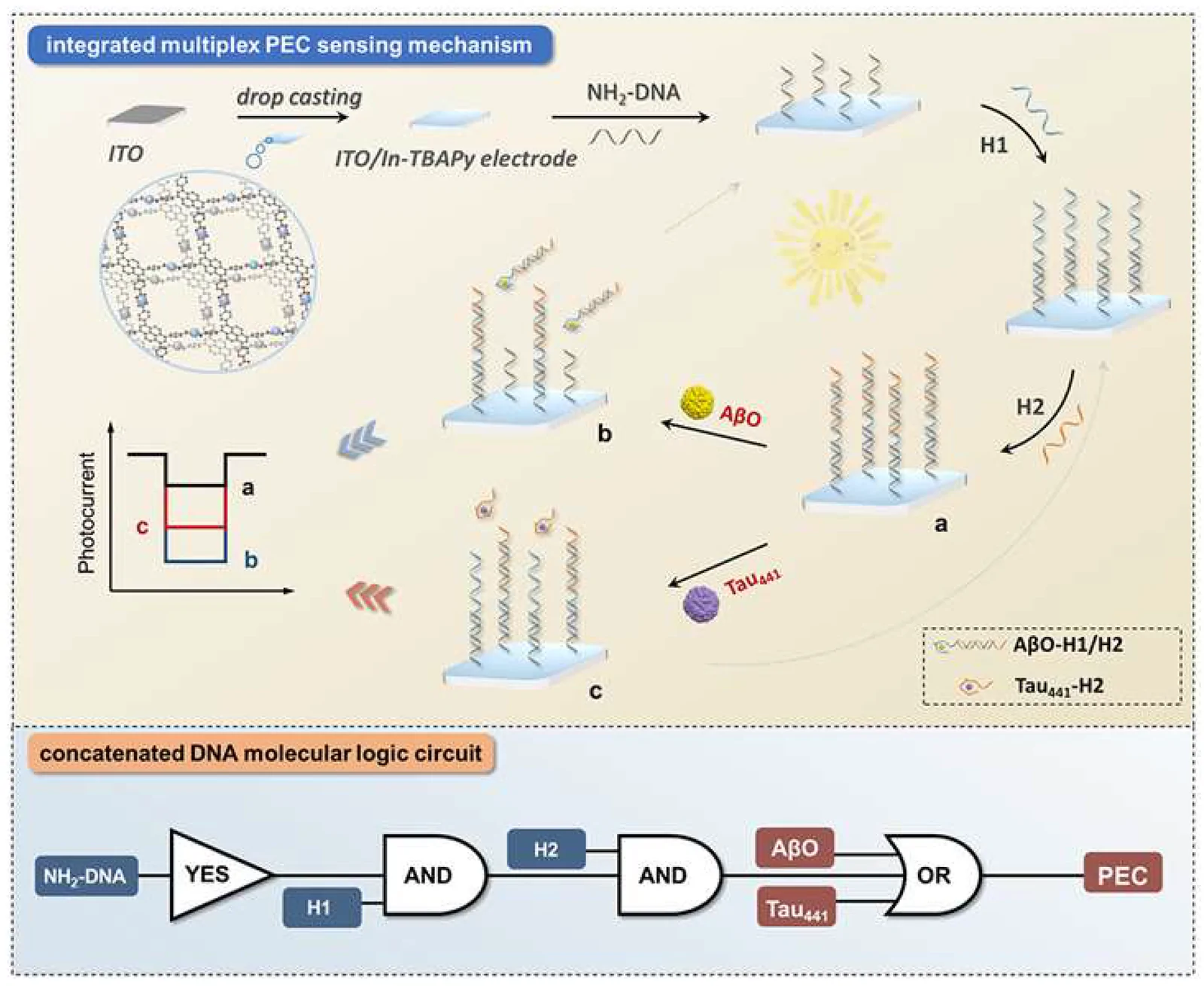
Schematic illustration of (**top**) the integrated multiplex PEC sensing mechanism for AβO and (**bottom**) corresponding Tau 441 concatenated DNA molecular logic circuit. Reprinted with permission from ref. [195].

Nucleic acid-based amplification strategies have been adopted in multiple research fields, greatly promoting the development of immunosensors especially in sensitivity improvement. The nucleic acid-based amplification technologies used for PEC immunoassays mainly include HCR and nuclease-assisted RCA. They can amplify the photocurrent signals by increasing the number of DNA fragments, photosensitizers, and biomimetic enzymes. As a simple and high-efficiency amplification technique, HCR exhibits great application potential in PEC bioanalysis, since it can realize linear or exponential signal amplification by assembling thousands of auxiliary hairpin probes. RCA is another highly efficient amplification method based on oligonucleotide templates. Under DNA polymerase catalysis, the amplification proceeds repeatedly in linear or geometric progression under isothermal conditions without thermal cycling and dedicated thermal cyclers. More importantly, the functionality of RCA products can be precisely and controllably tuned via rational design of circular template sequences to meet specific application demands. Gao et al. developed a rapid multiplex PEC aptasensor for the point-of-care detection of AβO and Tau441 based on a “signal off-on” mechanism (Figure 16) [195]. The aptasensor was built on the In-TBAPy MOF photocathode where the target binding detached the aptamer-analyte complex from the electrode surface, restoring the photocurrent. The key advancement of this work is the integration of a cascaded DNA OR logic gate, which can bypass time-consuming procedure and enable a fast YES/NO qualitative output. This logic-guided design not only simplified the fabrication procedure but also demonstrated the potential for intelligent rapid screening in home healthcare settings, representing a significant step toward practical point-of-care diagnostics for early assessment of AD risk.

## 6. QCM Biosensors

QCM is a widely used piezoelectric biosensor that can detect the changes in vibration frequency caused by mass changes resulting from target binding to the crystal surface. When quartz crystals bind to specific targets or antibodies, their vibration frequency will change, thus enabling target detection. To generate stable oscillation frequency, appropriate voltage is applied to the electrode on both sides of the crystal, producing shear waves parallel to the crystal surface. Crystal thickness, typically 5–20 MHz, is critical for oscillation frequency. In QCM systems, sensor performance is temperature-independent, and unlike electrochemical methods, there are no conductivity requirements for supporting materials. Additionally, QCM biosensors offer numerous advantages: signals can be analyzed via network analyzers, structures are simple, and frequency meters detect nanoscale mass changes [196]. A few groups have developed QCM biosensors to detect AD-related protein biomarkers [197,198]. For example, Wei’s group designed a sandwich dual-aptamer QCM biosensor for the detection of AβO and insoluble fibrils [198]. Aptamer-modified AuNPs were used to capture the targets and then injected into the aptamer-covered QCM chambers. This induced a significant frequency shift due to the large weight of AuNPs. The method showed a linear range of 0.2~10 pM for AβO with a detection limit of 0.11 pM, while the linear range for Aβ fibrils was 0.1~10 pM with a detection limit of 0.02 pM. However, the practical application ability of this biosensor was only investigated by spiking different concentrations of AβO into the artificial CSF samples.

## 7. FET and OECT Biosensors

**Figure 17:**
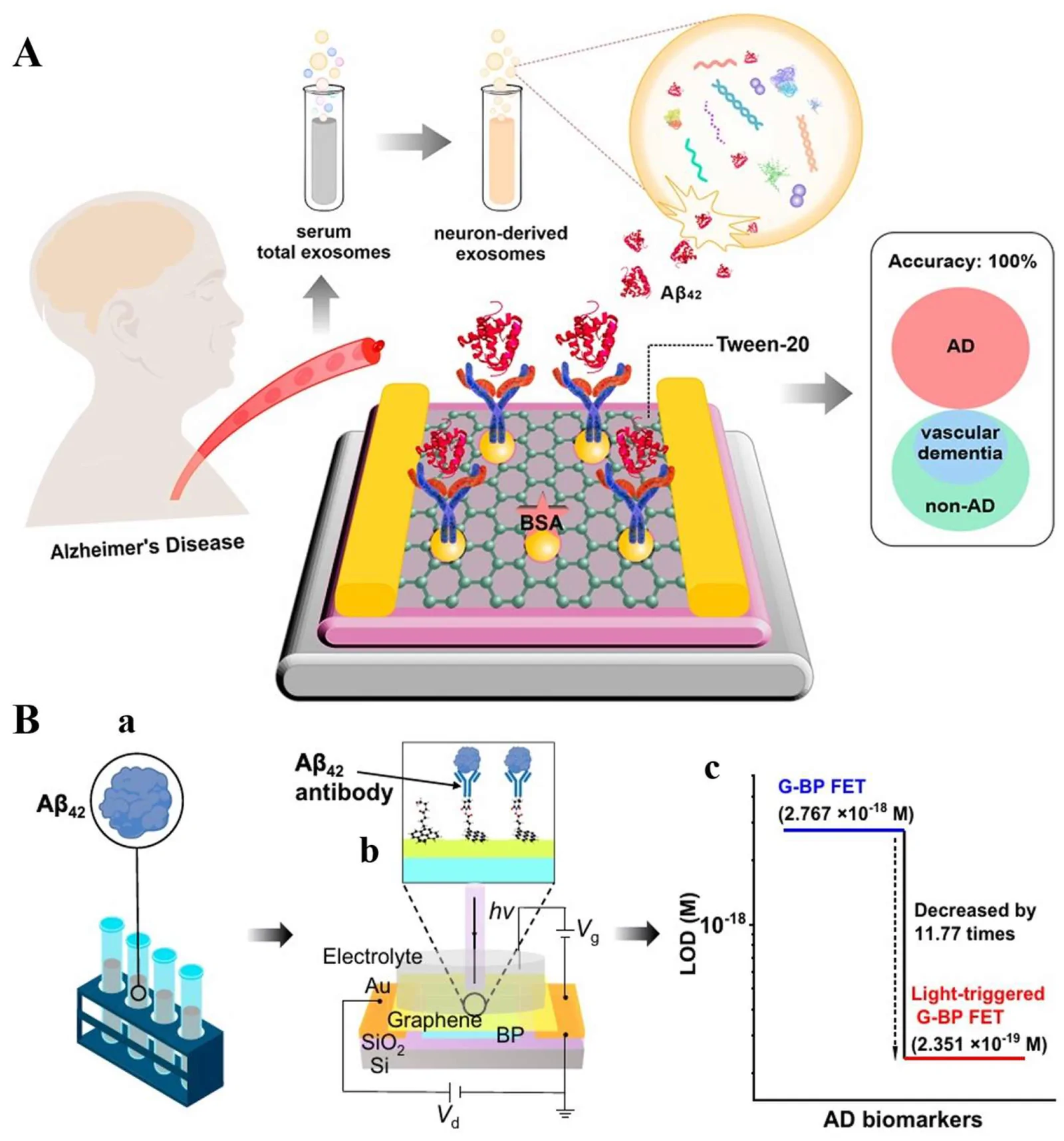
(**A**) Schematic illustration of the G-EGT biosensor for detection of NDE-Aβ42 in AD patients. Reprinted with permission from ref. [208]. (**B**) Schematic illustration of (**a**) Aβ42 antigen, (**b**) structural and principle of light-triggered G-BP FET biosensor, and (**c**) effect of light on the detection limit for Aβ42 protein. Reprinted with permission from ref. [209].

FET biosensors are mainly composed of two parts: components related to the field-effect transistor such as the channel material, and biologically functional components such as receptors [199]. The function of channel material is to convert the interaction between the target substance and the signal into an electrical signal, while receptors endow FET biosensors with the ability to specifically recognize targets in complex environments. In addition, the composition, morphology, structure, and physical and chemical properties of the biosensors also play important roles in their functions. The characteristics of the channel material and receptors directly affect the performance of FET biosensors for the detection of AD-related blood protein biomarkers [200,201,202,203,204,205,206,207]. The current response mainly depends on the changes in the number of electrons on the surface of the channel material, resulting from the interactions between charge carriers and receptors or proteins. When the target has the same charge polarity, electrostatic repulsion occurs, reducing the electron concentration on the channel surface and decreasing the current after target binding. Conversely, opposite charge polarity will lead to an increased current. When the receptor binds to the protein target in direct contact with or close to the channel material, charge from the receptor/target will transfer to the channel surface, altering electron density and generating a measurable current change. Graphene, with high carrier mobility (>10^5^ cm^2^ V^−1^ s^−1^), low sheet resistance, good biocompatibility, abundant modification sites, and ultra-sensitive response to electric fields and external doping, is the preferred FET material for biosensing. Li et al. developed an electrolyte-gated FET (G-EGT) based on AuNPs-modified graphene for early diagnosis of AD (Figure 17A) [208]. The method could sensitively determine exosomal Aβ42 in serum with a detection limit of 447 ag/mL. Clinical analysis of 27 subjects revealed the significant diagnostic value of Aβ42, reliably distinguishing AD from non-AD patients and even distinguishing AD from vascular dementia patients with 100% accuracy. This non-invasive, blood-based sensor holds great potential for personalized healthcare, suitable for advanced diagnosis and remote health monitoring. Recently, Luo’s group designed a light-activated graphene-black phosphorus (G-BP) heterojunction FET biosensor to tackle the bottleneck problems of insufficient sensitivity and severe matrix interference of traditional blood biosensors for trace plasma Aβ42 (Figure 17B) [209]. Graphene serving as the high-mobility conductive sensing channel showed a large specific surface area for antibody immobilization with few-layer black phosphorus as the light-responsive photoactive layer. The heterojunction structure resulted in synergistic electrostatic-photogating dual signal amplification, which is the core design to drastically lower the detection limit and overcome the Debye length screening effect in physiological buffer. Under dark conditions, specific binding of the negatively charged Aβ42 to its antibody triggered electrostatic gating and raised channel current. The method showed a detection limit of 2.767 aM for Aβ42. Upon illumination, BP produced massive photogenerated carriers to strengthen band bending, further amplifying the current response and pushing the detection limit down to 235.1 zM, three orders of magnitude superior to that of most existing biosensors. The platform featured excellent cycling and long-term stability, fast signal response (~300 s total detection time), and outstanding anti-interference capacity (almost no response to Aβ38 and Aβ40 analogues). When applied to serum samples, the biosensor maintained reliable linear response toward Aβ42, with a detection limit of 1.932 aM under light irradiation. This is indicative of its capability to quantify ultra-low concentrations of biomarkers in complex biological matrices and great promise for minimally invasive early screening and monitoring of diseases.

**Figure 18:**
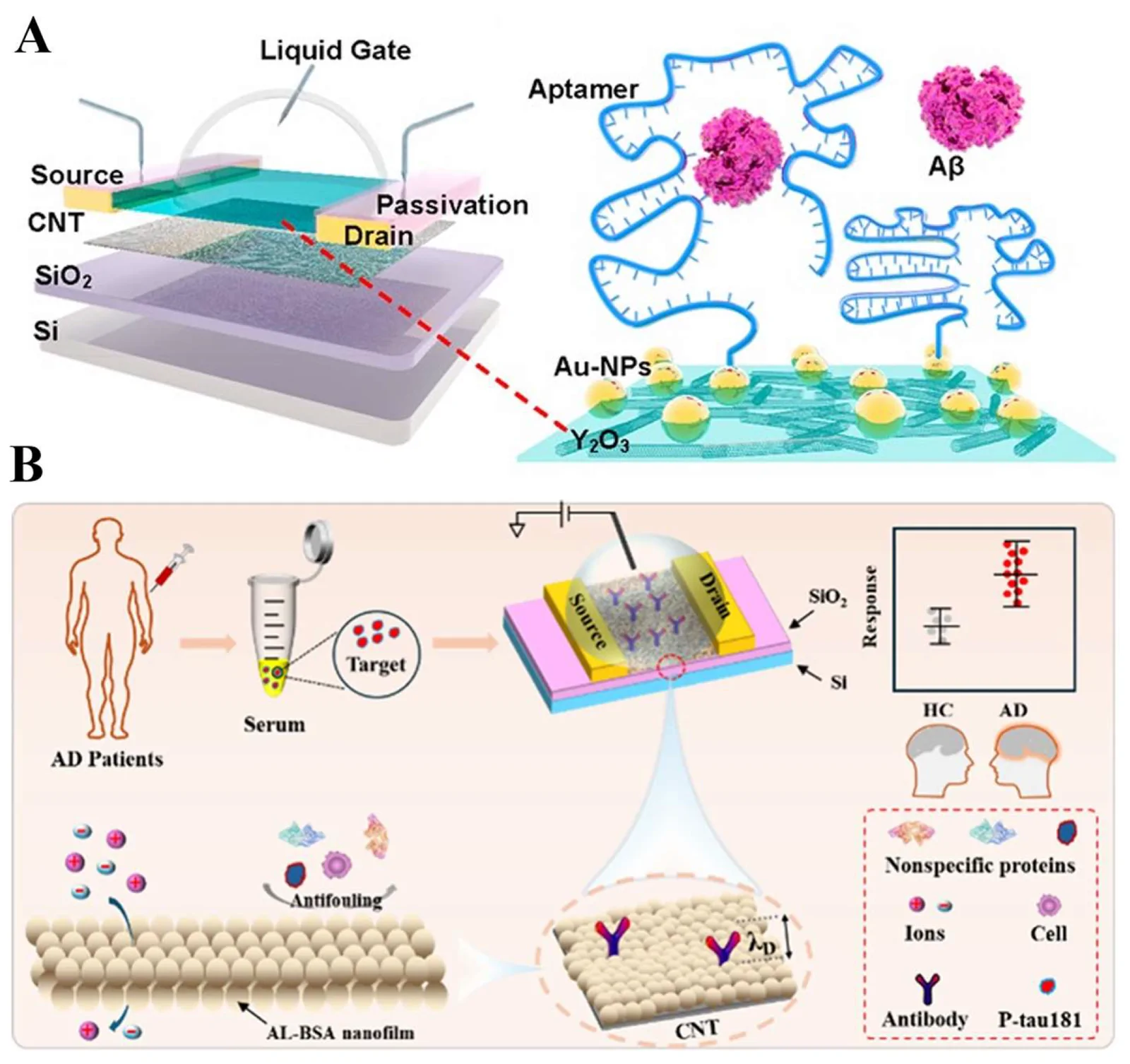
(**A**) Schematic illustration of aptamer-functionalized CNT-FET biosensor for the detection of AD serum biomarkers. Reprinted with permission from ref. [210] (**B**) Schematic illustration of amyloid-like-FET biosensor in the detection of p-Tau181 in human serum. Reprinted with permission from ref. [211].

Carbon nanotube-based FET (CNT-FET) is considered to be the most significant transistor technology beyond Moore’s era. In order to improve the antifouling ability and sensitivity of FET biosensors, Chen et al. developed an aptamer-functionalized FET biosensor based on highly uniform semiconductor CNT films for sensitively and selectively determining the core blood biomarkers of AD (Figure 18A) [210]. Thiolated DNA aptamers were immobilized on sensor channels via the deposited AuNPs. MCH, Tween 20, and bovine serum albumin were used to block non-specific adsorption. The target binding of Aβ and aptamer changed the conformation of DNA from folded to stretched structure, moving negatively charged aptamer regions away from CNT channels, reducing the current and shifting the transfer curve. The CNT-FET biosensor could reliably determine femtomolar level of Aβ40 and Aβ42 in whole human serum. Multiple blocking steps significantly reduced non-specific adsorption, improving the selectivity as high as 730% (Aβ40) and 800% (Aβ42). In addition, Wang et al. developed a CNT-FET biosensor for p-Tau181 detection with amyloid-like porous nanofilm as the intermediate layer (Figure 18B) [211]. The porous structure of the ovine serum albumen nanofilm increased the electrical stability and antifouling capability of the biosensor. The detection limit was found to be 0.1 fg/mL with almost 100% diagnostic accuracy for the assays of serum samples collected from 20 patients with AD and 5 patients without AD.

**Figure 19:**
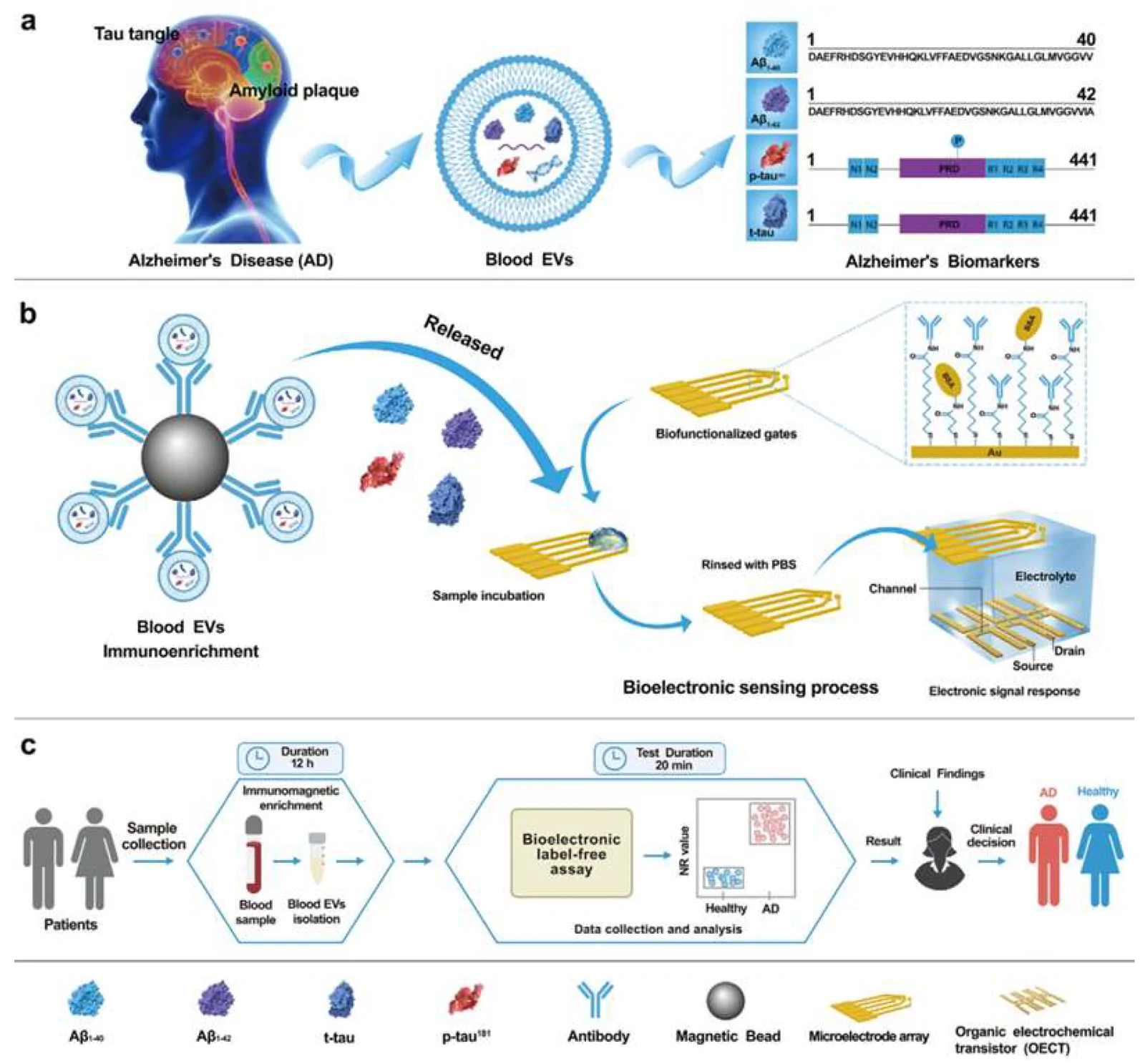
Schematic illustration of bioelectronic label-free platform for detecting core AD biomarkers in blood EVs. (**a**) Clinical diagnosis of AD using blood EVs-derived biomarkers. (**b**) Overview of the sensing process. (c) Workflow timeline from sample collection to result analysis. Reprinted with permission from ref. [215].

OECT is a biocompatible device that has become a promising biosensing technology due to its ability to detect and amplify signals in aquatic environments [212,213,214]. They can be performed by coupling electronic circuits with ion circuits through organic mixed-ion-electronic semiconductors as channel materials. Zheng et al. designed an OECT biosensor integrated with microelectrode arrays to overcome the limitations of low sensitivity and single-target detection (Figure 19) [215]. The platform was engineered for label-free multiplexed detection of four core AD biomarkers in blood extracellular vesicles (EVs) by using 2-(3,3′-bis(2-(2-(2-methoxyethoxy) ethoxy) ethoxy)-[2,2′-bithiophen]-5)yl thiophene p(g2T-T) organic semiconductor channels to amplify the signals. The change in interfacial capacitance induced by the attachment of target proteins on the antibody-functionalized hybrid SAMs modulated channel conductance, enabling ultrasensitive single-molecule detection without additional labeling. The platform achieved the quantitative analysis of Aβ40, Aβ42, total Tau, and p-Tau181 within only 20 min with zeptomolar detection limits and exhibited outstanding anti-fouling performance in serum matrices. An AUC of 0.965 was attained for Aβ42 detection on 40 sets of clinical blood EV samples. The combined multivariate analysis of the four biomarkers achieved 100% classification accuracy to distinguish AD from healthy controls, outperforming traditional ELISA. This EV-based multi-index bioelectronic sensing strategy provided a minimally invasive, high-precision scheme for early screening of AD and monitoring of longitudinal disease progression.

In summary, electrochemical biosensors are the most mature and versatile platform for AD-relative blood biomarker detection. However, they are susceptible to biofouling in whole blood, often requiring sample pre-treatment or antifouling coatings. Although direct detection methods with label-free characteristic are simple and rapid, they often show limited sensitivity and high background in complex blood samples. In contrast, sandwich-type immunoassays exhibit more sensitive and specific and yet require complicated operation steps and high cost. ECL biosensors provide ultrahigh sensitivity and are the preferred choice for centralized clinical laboratories. Nevertheless, their dependence on expensive luminophores and photomultiplier tubes limits their wide application in resource-limited settings. PEC biosensors offer the advantage of dual energy forms (light excitation and current readout), which drastically reduces background noise and enables detection limits comparable to ECL. However, the stability of photoactive materials under prolonged irradiation and in biological media remains a concern. FET and OECT biosensors have become ideal for wearable and point-of-care applications, but their fabrication reproducibility and long-term stability still lag behind conventional platforms. Recently, a number of emerging biosensors are beginning to reshape the landscape of these established FET and OECT biosensors and hold considerable promise for future AD diagnostics such as photoelectrochemical transistors (PECTs) [216]. In addition, electrical transistors with in-sensor computing systems have been reported for real-time detection of biomarkers without analog-to-digital conversion and external data processing units [217]. Flexible neuromorphic electronics have been developed for wearable in-sensor health monitoring, enabling real-time on-body collection, processing, and decision-making. Although these technologies have not yet been specifically applied to determine protein biomarkers of neurodegenerative diseases, their underlying principles and architectures are readily adaptable to AD diagnostics.

## 8. Conclusions and Future Perspectives

AD has become a severe challenge faced by humanity in the global public health field, posing a significant socioeconomic burden. Traditional diagnostic methods such as CSF analysis and Aβ-PET or Tau-PET neuroimaging still exhibit some insurmountable limitations, including invasiveness and high cost. Blood biomarker detection offers a minimally invasive, convenient, and reproducible solution for the early diagnosis and prognostic assessment of AD. It is poised to overcome the drawbacks of currently used testing and imaging procedures in terms of invasiveness, cost, and equipment dependency, thereby reshaping the landscape of AD diagnosis and treatment. However, the extremely low concentration of protein biomarkers in AD blood, coupled with the complexity of blood components, poses severe challenges to the sensitivity, specificity, and anti-interference capabilities of biosensors. This review systematically summarizes the recent advance of electrochemical and electrical biosensors in the detection of AD-related blood biomarkers, with a focus on detection modes and signal amplification strategies. Although high-affinity antibodies remain the mainstream recognition elements, aptamers and MIPs are emerging as powerful complements due to their excellent thermal stability, batch reproducibility, and low cost, potentially overcoming the cross-reactivity issues of antibodies in complex samples. Nanomaterials such as gold/silver nanoparticles, carbon nanotubes, graphene, and MOFs are widely used for electrode modification to increase effective surface area and facilitate electron transfer. Simultaneously, signal amplification strategies such as enzyme-catalyzed precipitation, nucleic acid amplification, and CRISPR/Cas systems further push the detection limit to fg/mL or even ag/mL levels. More importantly, an increasing number of methods have begun to apply the constructed sensors to clinical serum or plasma samples from patients, with results consistent with traditional clinical diagnostic methods.

Despite significant advancements in electrochemical and electrical biosensors for the detection of AD biomarkers, there are still several bottlenecks that urgently need to be overcome. First, the concentration of AD biomarkers in blood is very low due to the blood-brain barrier, and the components of blood samples are complex. Although the reported biosensors are highly sensitive in serum or plasma samples, the sensing interface is susceptible to non-specific adsorption and biofouling, which severely reduces reproducibility and long-term stability. The interference in blood may damage the biorecognition elements of the sensing interface, further leading to the false signal. Second, blood biomarkers exhibit inherent biological heterogeneity, and relying solely on the concentration changes of a single biomarker is difficult to comprehensively reflect the complex pathological processes and individual differences of AD. Currently, research on multi-target joint detection is still very weak. Third, there is a lack of unified operational standards for pre-analysis steps such as blood sample collection, processing, preservation, and transportation, and minor differences can significantly affect the stability and reliability of detection results. At the same time, the sensor preparation process also lacks a standardized quality control system. Differences in electrode modification, recognition element immobilization, and test parameter settings among different laboratories make it difficult to directly compare and reproduce experimental results across different platforms and laboratories. Fourth, most current methods are validated only through spiked recovery experiments using 1-3 normal serum samples, lacking systematic evaluation in large-scale, multi-center clinical cohorts, making it difficult to objectively reflect their true discriminatory performance in heterogeneous populations. Fifth, most current sensors are still in the proof-of-concept stage, lacking deep integration with automated sample pre-treatment, intelligent data analysis, and cloud-based health management platforms.

Several emerging directions or tasks are expected to break through the aforementioned bottlenecks and propel the field towards clinical practical application. First, designing microfluidic chips integrated with sample pre-processing functions to automatically complete blood separation, biomarker enrichment, and reaction cleaning can effectively reduce non-specific adsorption and environmental interference. Introducing novel signal amplification strategies such as CRISPR/Cas systems, aptamers, or proximity extension assay can push sensitivity to the femtomolar level. MIPs, DNA nanostructures, and aptamers replacing traditional antibodies can not only improve thermal stability and batch reproducibility, but also effectively avoid cross-reactivity in complex samples. Second, it is promise to simultaneously quantify multiple core biomarkers in a single test, such as Aβ42/Aβ40 ratio, p-Tau217, p-Tau181, NFL, and GFAP, by constructing the detection platforms combining microfluidic chips and array electrodes. On this basis, by combining AI algorithms for fusion analysis of multiplex biomarker data, establishing disease classification and risk prediction models can promote the transition from “single biomarker detection” to “multi-dimensional molecular profiling analysis”. Third, it is necessary to unify pre-analysis operational specifications for blood sample to minimize systematic differences between batches and laboratories. The quality control standards for sensor preparation, unifying electrode modification processes, recognition element immobilization methods, and test parameter settings can ensure consistency in sensor performance across different laboratories and batches. Developing standardized reference materials and calibrators may provide a material basis for mutual recognition and traceability of results across different platforms. Fourth, it is crucial to address the lack of large-scale clinical validation and conduct multi-center, prospective large-scale clinical cohort studies, which can clarify their positioning and value in different clinical scenarios. Strengthening cooperation between laboratories and hospitals may facilitates the actual efficacy evaluation of electrochemical and electrical biosensors in early screening, differential diagnosis, and disease staging in real clinical scenarios. It is important to compare the detection results of novel biosensors with established immunoassay methods (e.g., ELISA and single molecule immunoassay), CSF biomarker analysis, and PET neuroimaging techniques. Fifth, it is prevalent to develop intelligent sensing systems integrated with automated sample pre-processing to achieve full-process automation from sample loading to result output. By combining ECT, OPECT, or PECTs with sensing and computational capabilities with wearable devices, researchers can construct multimodal diagnostic models that integrate blood biomarkers, cognitive assessment, and wearable behavior data, truly realizing the transformation from laboratory research to clinical decision support. Combined with smartphone readers and wireless data transmission, this will provide a low-cost and easy-to-operate detection platform, suitable for primary care and home self-examination scenarios.

## Figures and Tables

**Table 1:** Comparison on the performance of different electrode materials.

Materials	Conductivity	Stability	Applicability	Advantage	Limitation
Metal	Excellent	Good	High	High conductivity; facile surface functionalization; large surface area; enhanced electron transfer rate	AgNPs prone to oxidation and deactivation; batch-to-batch variation; potential toxicity from metal ion leakage
Carbon	Excellent	Good	High	Ultra-high surface area; excellent conductivity; functionalizable (oxygen-containing groups)	Strong non-specific adsorption; dispersion challenges; sharp edges may cause mechanical damage to cell membranes
MOFs	Moderate	Moderate	Moderate	Ultra-high surface area; tunable pore size; abundant binding sites; capability to encapsulate signal molecules	Some MOFs lack water stability and conductivity requires improvement through the formation of composites
SAMs	Weak	Good	Moderate	Controllable and highly ordered interface; Low non-specific adsorption; tunable terminal groups	Poor conductivity of long-chain SAMs; complex fabrication procedure; limited long-term stability
Polymers	Good	Moderate	Moderate	Excellent flexibility; good biocompatibility; solution processability	Poor long-term stability; conductivity depending on doping state

## Data Availability

No new data were created or analyzed in this study. Data sharing is not applicable to this article.
